# Cellular Membrane‐Targeted Europium(III) Probe for Selective and Time‐Resolved Detection of Peroxynitrite

**DOI:** 10.1002/chem.71463

**Published:** 2026-07-27

**Authors:** Jamie Webb, Rebecca Tiffany, Karolis Norvaisa, Hennie Valkenier, Lea Miebach, Sander Bekeschus, Stephen J. Butler

**Affiliations:** ^1^ Department of Chemistry Loughborough University Loughborough UK; ^2^ Engineering of Molecular Nanosystems, Ecole Polytechnique De Bruxelles Université Libre de Bruxelles (ULB) Brussels Belgium; ^3^ ZIK Plasmatis Leibniz Institute for Plasma Science and Technology (INP) Greifswald Germany; ^4^ Department of Hematology and Oncology Greifswald University Medical Center Greifswald Germany; ^5^ Department of Dermatology, Venerology, and Allergology Rostock University Medical Center Rostock Germany

**Keywords:** activity‐based probe, lanthanide probe, luminescent sensing, peroxynitrite, reactive oxygen species

## Abstract

Peroxynitrite (ONOO^−^) is an extremely short‐lived and highly reactive species whose biological effects depend on its local concentration at the site of formation. Consequently, detecting and quantifying ONOO^−^ requires probes that combine high selectivity with well‐defined subcellular localization. The cell membrane is a particularly important yet underexplored interface, as it is the first barrier encountered by extracellularly generated ONOO^−^ and determines how much of this species penetrates into the cell. Such membrane‐proximal ONOO^−^ formation occurs during inflammatory processes and oxidative stress–based therapies, and is especially relevant in cold atmospheric plasma treatment, where complex RONS mixtures interact with the membrane before intracellular effects arise. Here, we report **Eu.Chol**, a luminescent europium(III) complex that integrates a phenylboronic acid ONOO^−^ reactive motif with a cholesterol anchor to localize the probe within lipid bilayers. We demonstrate that **Eu.Chol** incorporates efficiently into liposomes and Jurkat cells, enabling selective, membrane‐localized ONOO^−^ detection with minimal response to H_2_O_2_. These results establish a modular europium(III)‐based scaffold for targeted ONOO^−^ sensing, enabling potential incorporation of organelle‐specific targeting groups to probe RONS dynamics at defined sub‐cellular sites.

## Graphical abstract

We report a cellular membrane‐targeted europium(III) probe, **Eu.Chol**, that enables selective, time‐resolved detection of peroxynitrite (ONOO^−^) at the cell membrane interface. **Eu.Chol** demonstrates efficient incorporation into liposomes and Jurkat cells, with minimal response to H_2_O_2_, establishing a modular platform for subcellular ONOO^−^ sensing.

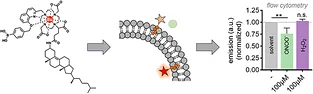

## 1 Introduction

Peroxynitrite (ONOO^−^) is a highly reactive nitrogen species formed from the rapid reaction of superoxide (O_2_^−^) with nitric oxide (NO), predominantly in the mitochondria, where it participates in signaling through tyrosine nitration and protein thiol nitrosylation [1, 2, 3, 4]. At elevated levels, ONOO^−^ causes oxidative and nitrosative damage to lipids, proteins, and DNA, and is implicated in neurodegenerative diseases such as Alzheimer's, Huntington's, and Parkinson's [5, 6, 7]. Detecting ONOO^−^ with selectivity over a range of reactive oxygen and nitrogen species (RONS) is therefore critical for understanding its biological roles, improving disease diagnostics, and developing therapies that exploit redox signaling, including photodynamic and radiotherapy [8].

Cellular membranes play a central role in regulating enzymatic activity, signaling, and ion transport [9]. The plasma membrane, in particular, acts as the primary interface for extracellular reactive species [10], including RONS generated by cold atmospheric plasma (CAP) therapies [10, 11]. CAP is emerging as a promising modality for the treatment of chronic wounds and several cancers [12, 13, 14]. CAP's biological effects are thought to be mediated mainly by RONS, including ONOO^−^, which can disrupt bacterial biofilms or selectively kill tumor cells by overwhelming antioxidant defences [15, 16, 17, 18]. However, the mechanisms by which plasma‐generated RONS interact with the cell membrane, cross into the cytosol, or initiate signaling remain poorly understood [19, 20].

Detecting ONOO^−^ is challenging due to its short lifetime (10–20 ms) [21] in biological environments and rapid conversion to nitrate. A common indirect detection strategy involves immunostaining of 3‐nitrotyrosine, a stable marker of protein nitration; however, this approach is restricted to fixed samples and is incompatible with dynamic or live‐cell studies. Several fluorescent probes have been developed to detect ONOO^−^, often based on phenylboronic acids and eliciting an ‘off–on’ emission response [22, 23, 24, 25, 26]. While some of these probes show significant promise by enabling ONOO^−^ detection in living cells, many still suffer from short‐lived fluorescence signals that overlap with cellular autofluorescence, low water‐solubility, or limited selectivity, reducing measurement reliability and precluding quantitative imaging in complex biological environments. Notably, although numerous probes have been developed for sub‐cellular imaging of ONOO^−^ within organelles such as mitochondria, lysosomes, and the Golgi apparatus [27], examples capable of interrogating ONOO^−^ at the cellular interface, particularly at the plasma membrane, remain rare.

Lanthanide(III)‐based luminescent probes are particularly attractive for biological sensing and imaging due to their long‐lived excited states, which enable time‐gated detection with high signal‐to‐noise ratios, and their sharp, line‐like emission bands that allow selective observation within narrow spectral windows [28, 29, 30, 31, 32, 33]. Importantly, the design of the ligand architecture surrounding the lanthanide(III) ion enables selective and sensitive recognition of biologically relevant anions [34, 35, 36, 37, 38, 39, 40], cations [41, 42], biomolecules [43, 44, 45], and reactive species [46, 47, 48] by modulating coordination geometry, hydration state, and energy transfer pathways. In this context, our previously reported Eu(III) probe **Eu.1** (Figure 1) uses these photophysical and ligand design features to achieve time‐resolved detection of ONOO^−^ with low micromolar sensitivity in living cells [49]. The probe features a quinoline antenna bearing a peroxynitrite‐reactive phenylboronic acid, which undergoes oxidative cleavage to reveal an 8‐hydroxyquinoline, quenching the Eu(III) emission. This probe was able to penetrate living cells and preferentially accumulate in the mitochondria due to its inherent localization profile, rather than through deliberate targeting, enabling ONOO^−^ detection following CAP treatment. However, it was limited in its ability to quantify ONOO^−^ at different cellular sites, motivating us to develop a next‐generation probe design with explicit organelle or membrane‐targeting functionalities.

**FIGURE 1:**
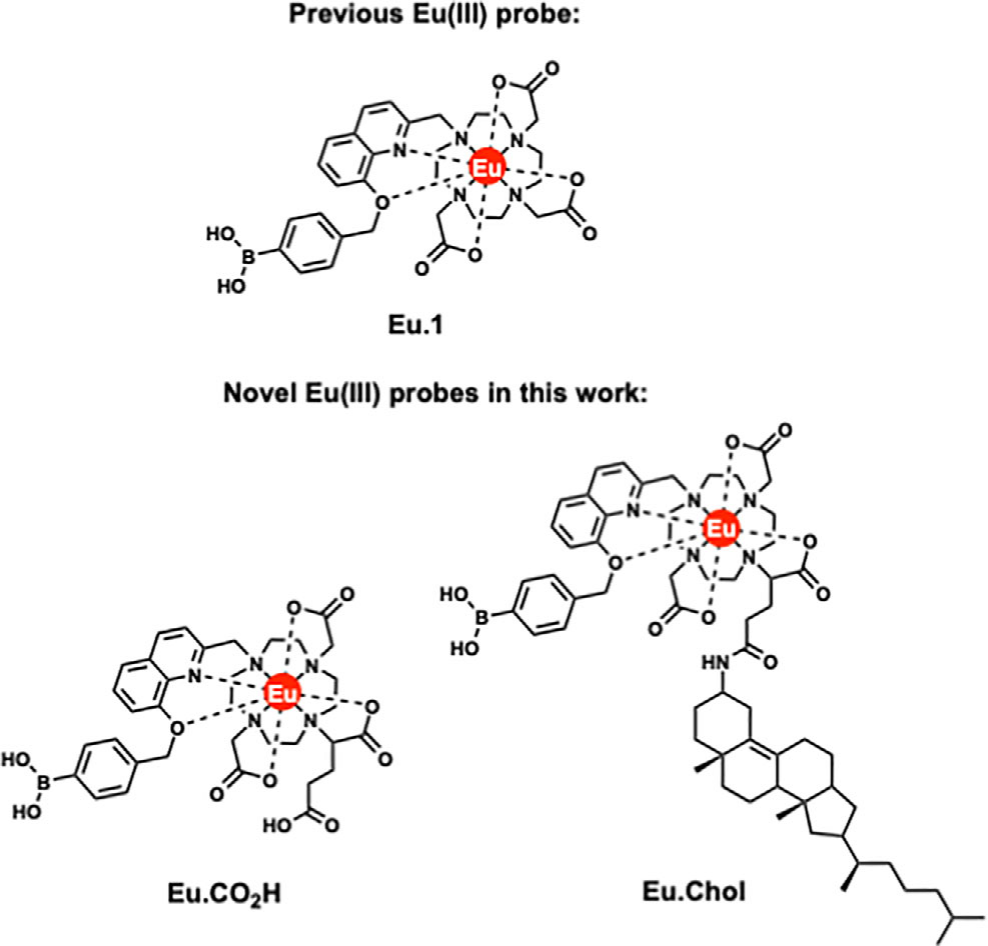
Structure of previously reported Eu(III) probe **Eu.1** [49] and novel probes **Eu.CO_2_H** and **Eu.Chol** presented in this work.

Accordingly, here we introduce **Eu.Chol** (Figure 1), an overall neutral Eu(III) probe built on a modular **Eu.CO_2_H** scaffold, combining a phenylboronic acid ONOO^−^‐reactive motif with a cholesterol unit to promote membrane localization. **Eu.Chol** exhibits high selectivity for ONOO^−^ over other reactive species, excellent sensitivity, and an instantaneous luminescence response, making it a robust reporter of ONOO^−^ dynamics. Liposome studies confirmed incorporation of **Eu.Chol** into lipid bilayers, while Jurkat cell assays demonstrated rapid membrane staining and a selective ONOO^−^ response over H_2_O_2_. These results show how the modular **Eu.CO_2_H** scaffold can be adapted toward sub‐cellular ONOO^−^ sensing, exemplified here by **Eu.Chol**, which enables interrogation of RONS reactivity at the membrane of living cells.

## 2 Results and Discussion

### 2.1 Probe Design Considerations

The novel europium(III) complexes **Eu.CO_2_H** and **Eu.Chol** (Figure 1) are built on a common macrocyclic scaffold comprising a cyclen core, with a coordinating quinoline pendant arm that binds Eu(III) in a bidentate manner. This configuration effectively shields the metal centre from water, enhancing both quantum yield and emission lifetime. Three pendant carboxylate arms confer high kinetic and thermodynamic stability, one of which is derived from glutamic acid and terminates in a free carboxylic acid in **Eu.CO_2_H**, providing a functional handle for sub‐cellular targeting. The quinoline arm serves a dual purpose: it sensitises Eu(III) emission and bears a peroxynitrite‐reactive phenylboronic acid, which undergoes oxidative cleavage to quench Eu(III) emission *via* charge transfer or intramolecular proton transfer.

**Eu.Chol** is generated by amide coupling of a cholesterol unit to the glutamic acid carboxylate, promoting localisation within lipid bilayers and targeting the cell membrane. This design enables direct monitoring of ONOO^−^ transport and interactions at the cell–surface interface. Cholesterol derivatives are well known to increase lipophilicity and facilitate stable membrane association [50, 51], and **Eu.Chol** relies on the quinoline antenna to sensitise Eu(III) emission, allowing direct detection of ONOO^−^ levels at the membrane interface *via* time‐resolved luminescence. Each Eu(III) complex was built in a modular fashion from a common **Eu.Iodo** scaffold (Scheme 1), which enables late‐stage functionalisation with both the boronic acid and sub‐cellular targeting unit, thereby providing a route toward the development of a diverse probe library.

**SCHEME 1:**
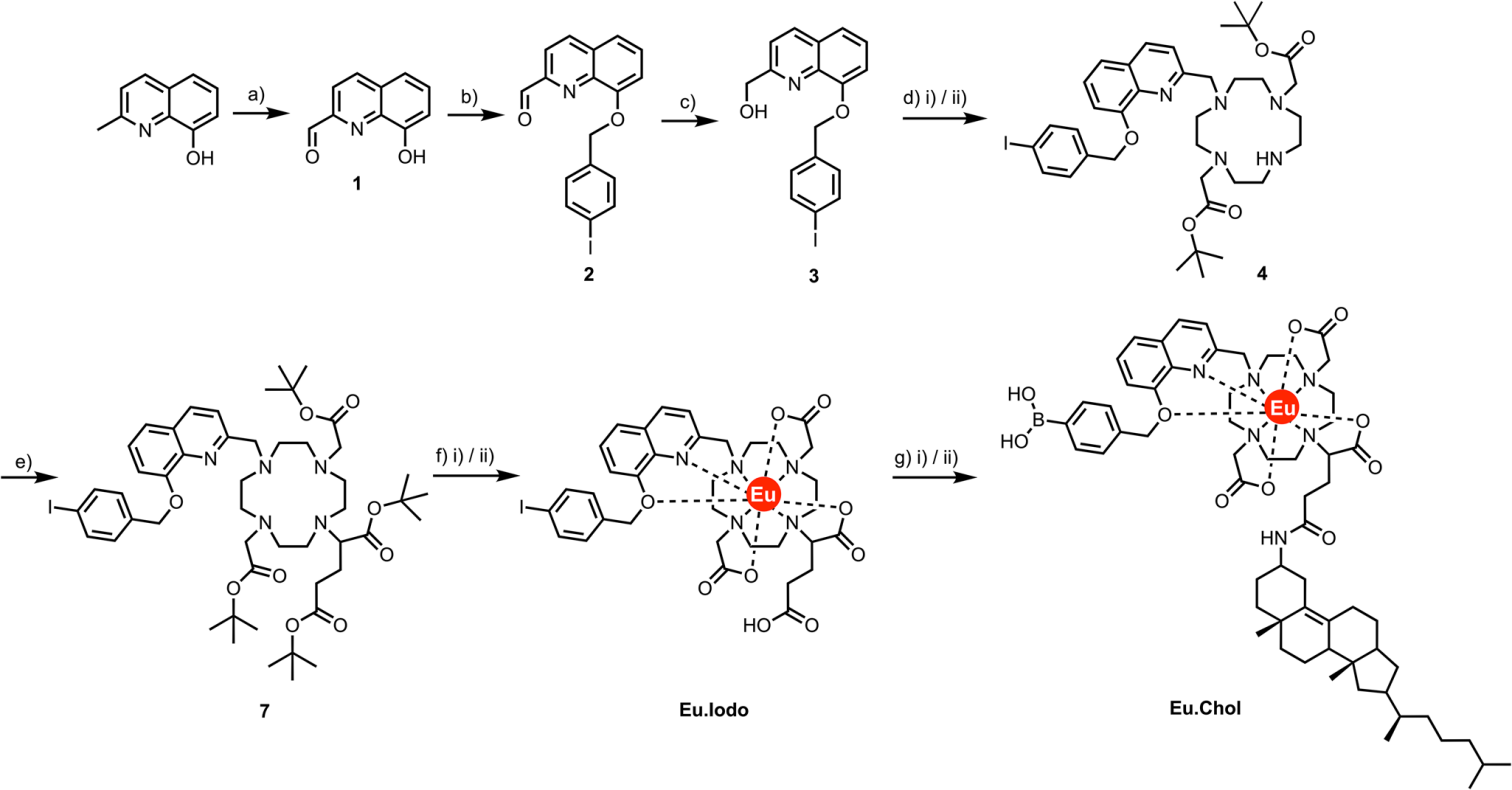
Synthesis of europium(III) complex **Eu.Iodo**, which was subsequently used to prepare **Eu.Chol** and **Eu.CO_2_H**. Conditions: (a) 1,4‐dioxane, SeO_2_, 80°C, 60%; (b) BrCH_2_PhI, K_2_CO_3_, CH_3_CN, r.t., 68%; (c) NaBH_4_, MeOH, r.t., 98%; (d) (i) MsCl, triethylamine, THF, r.t.; (ii) DO2A‐*tert‐*butyl ester, K_2_CO_3_, CH_3_CN, 70°C, 29%; (e) **6**, K_2_CO_3_, KI, CH_3_CN, 70°C, 63%; (f) (i) TFA/DCM 50/50; (ii) EuCl_3_∙6H_2_O, H_2_O, 50°C, quant 100%; g) (i) **9**, HATU, DIPEA, DMF, r.t., 14%; (ii) B_2_pin_2_, Pd(dppf)Cl_2_, KOAc, MeOH, 60°C, 42%.

### 2.2 Synthesis

Full details of the synthesis of **Eu.Chol** and **Eu.CO_2_H** are provided in the Supporting Information. Both complexes were prepared from the common **Eu.Iodo** scaffold (Schemes 1 and S1). First, a Riley oxidation of 8‐hydroxy‐2‐methylquinalidine was conducted to produce the aldehyde **1** which was then *O*‐alkylated with 4‐iodobenzyl bromide to give intermediate **2**. A reduction of the aldehyde to the primary alcohol **3** followed. Subsequent mesylation of alcohol **3** produced the methanesulfonate ester which was then used for the alkylation of *tert*‐butyl‐protected DO2A to afford the macrocyclic compound **4**. The modest yield (29%) reflects the challenge of controlling mono‐alkylation and competing bis‐alkylation; this was mitigated by using 0.9 equivalents of mesylate ester and careful LC–MS monitoring to limit over‐alkylation. The α‐bromoester **6** (Scheme S1) was prepared from *L*‐glutamic acid in two steps and reacted with macrocycle **4** to give the protected ligand **7**. It is possible that partial racemisation of the alkylating agent **6** or racemisation during the reaction with **4** may have produced the protected ligand **7** as a mixture of stereoisomers; however, the stereogenic centre is remote from both functional domains of the probe. Specifically, the cholesterol unit is separated by a flexible linker and the ONOO^−^‐reactive benzyl boronic acid is located on the opposite side of the molecule, and therefore stereoisomerism is not expected to significantly influence the sensing or localisation behaviour under the conditions studied. The yield of this alkylation step to afford **7** was significantly improved by the addition of potassium iodide (K_2_CO_3_/acetonitrile), presumably *via* iodide/bromide exchange. Global deprotection of the *tert*‐butyl groups of **7** using trifluoroacetic acid revealed the free ligand, which was then reacted with one equivalent of EuCl_3_∙6H_2_O in water (pH 7.5) to afford the benzyl iodide scaffold **Eu.Iodo** as the chloride salt.

**Eu.Chol** was synthesised as shown in Scheme S2, *via* amide coupling between **Eu.Iodo** and cholesterol amine **9** to generate the precursor complex **Eu.Chol.Iodo**. The final phenylboronic acid group was installed through Pd(dppf)Cl_2_‐catalysed borylation of **Eu.Chol.Iodo** with bis(pinacolato)diboron in methanol. The borylation conditions were adapted from those reported by Barrosa and co‐workers [52] and first applied to **Eu.1**, which improved the yield from 59% to 87% by reducing the equivalents of potassium acetate from 6 to 4.5 and using methanol instead of DMSO, minimising the photodeborylation side‐product and allowing completion in under 1 h. These optimised conditions were subsequently applied to the synthesis of the novel complex **Eu.Chol**, which was purified by reverse‐phase chromatography under acidic conditions, eluting with 100% methanol. **Eu.CO_2_H**, which serves as a control lacking the peripheral cholesterol functionality, was prepared directly from **Eu.Iodo** under identical Pd(dppf)Cl_2_‐catalysed borylation conditions (Scheme S3) and purified by RP‐HPLC under acidic conditions, eluting with approximately 75% methanol.

### 2.3 Photophysical Analysis of Eu(III) Complexes

The photophysical properties of **Eu.Chol** and **Eu.CO_2_H** are summarised in Table 1, alongside the previously reported **Eu.1** [49]. Measurements were conducted in 100 mM PBS (pH 7.4), except for the more lipophilic **Eu.Chol**, for which an 80:20 PBS/MeOH mixture was used. Solubility studies established the minimum methanol content required to maintain stable absorption and emission spectra of **Eu.Chol** in 100 mM PBS (pH 7.4). **Eu.Chol** remained fully soluble in 2% methanol, with less than 5% variation in absorbance or emission intensity over 30 min. Based on these results, an 80:20 PBS/MeOH mixture was selected for subsequent studies to better approximate the polarity of the membrane interfacial region, which is less polar than bulk water. **Eu.Chol** and **Eu.CO_2_H** showed UV–Vis spectra similar to **Eu.1**, with a broad band centred at 322 nm (Figures 2 and S7). **Eu.Chol** (5,900 M^−^^1^ cm^−^^1^) displayed a 2‐fold increase in extinction coefficient compared with **Eu.CO_2_H** and **Eu.1**, which may be attributed to solvent polarity effects from the PBS/MeOH mixture.

**TABLE 1:** Photophysical data for Eu(III) complexes measured in 100 mM PBS (pH 7.4), unless stated otherwise.

Complex	λ_max_ (nm)	ε (M^−^^1^ cm^−^^1^)	*τ*_H_ (ms)^b^	*τ*_D_ (ms)^b^	*q* ^c^	Φ_em_ (%)^d^	B (M^−1^ cm^−1^)
**Eu.1** ^a^	322	2,800	0.52 ± 0.08	0.66 ± 0.08	0.19	4.5	126
**Eu.CO_2_H**	322	2,900	0.63 ± 0.09	0.78 ± 0.11	0.07	5.0	144
**Eu.Chol**	322	5,900	0.71 ± 0.11	0.78 ± 0.11	0.27	3.3	195

^a^ Photophysical values for **Eu.1** reported previously [49].

^b^ Lifetime measurements were conducted in duplicate with errors determined through the averages ± standard deviation. Lifetime measurements were conducted in methanol and deuterated methanol for **Eu.Chol**, and in water and D_2_O for **Eu.1** and **Eu.CO_2_H**.

^c^ Values of hydration state, *q* (±20%) were derived using literature methods [53, 54].

^d^ Quantum yield values represent the Eu(III)‐centred emission (550–720 nm) and were measured using quinine sulfate in 0.05 M H_2_SO_4_ as standard (*Φ*_em_ = 60%) [55].

Errors in quantum yields are ±15%. e The Eu(III)‐centred quantum yield of **Eu.1** was determined to be 4.5% using quinine sulfate in 0.05 M H_2_SO_4_ as a standard. This value is lower than the previously reported value of 10% [49], which was based on comparison with the structurally related complex **Eu.Bn** (*Φ*_em_ = 6%) reported by Williams and coworkers [56].

**FIGURE 2:**
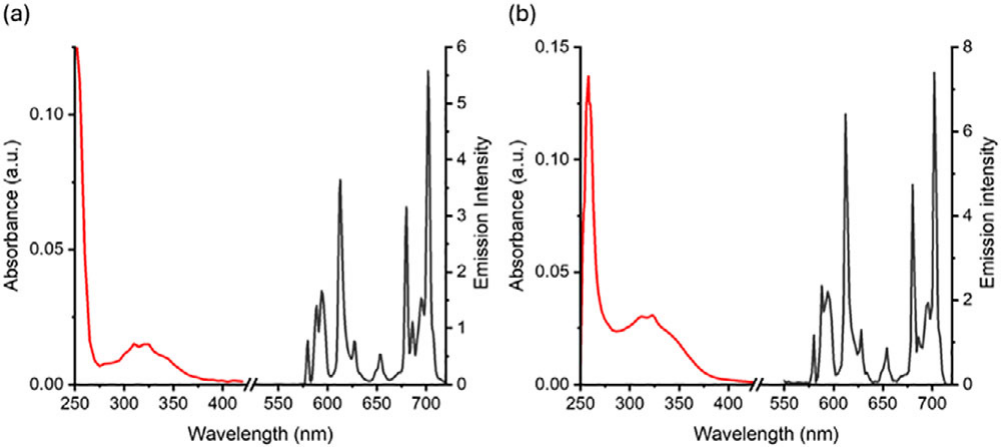
Absorption (red) and emission (black) spectra of (a) **Eu.CO_2_H**, 25 µM, *λ*_exc_ = 322 nm, 100 mM PBS buffer, pH 7.4, 295 K; (b) **Eu.Chol**, 25 µM, *λ*_exc_ = 322 nm, 80:20 100 mM PBS/MeOH, pH 7.4, 295 K.

Following excitation at 322 nm, novel complexes **Eu.CO_2_H** and **Eu.Chol** exhibited characteristic Eu(III) luminescence (Figure 2a,b), with negligible ligand‐centred fluorescence, confirming efficient energy transfer from the quinoline antenna. Their Eu(III)‐centred quantum yields (550–720 nm) were 5% and 3.3%, respectively, closely matching **Eu.1**. The emission spectral shape of **Eu.Chol** and **Eu.CO_2_H** was comparable to that of **Eu.1** (Figure S8), displaying a prominent feature in the Δ*J* = 2 band (605–630 nm) and a large Δ*J* = 4 band (680–705 nm), comprising four distinct peaks.

Within the Δ*J* = 1 (585–605 nm), three microstates are expected in principle, but two appear merged in the observed spectra (Figure 2). In the new complexes, this broader merged component is shifted to slightly higher energy, and the Δ*J* = 2 / Δ*J* = 1 intensity ratio is somewhat larger (approx. 1.5) than for **Eu.1** (approx. 1), while most other spectral features remain comparable. These subtle differences suggest that the glutamic acid arm in **Eu.Chol** and **Eu.CO_2_H** influences the coordination geometry around the Eu(III) centre; however, these effects are modest, as reflected by the fact that key photophysical parameters such as quantum yield and lifetime are not significantly changed.

The emission lifetime of **Eu.Chol** measured in methanol was longer than that of the control complexes **Eu.CO_2_H** and **Eu.1** measured in water (by approximately 12% and 36%, respectively). This difference is primarily attributed to reduced vibrational quenching from O–H oscillators in methanol compared with water [53]. In addition, the bulky cholesterol substituent may contribute to additional shielding of the Eu(III) centre. The longer lifetime of **Eu.CO_2_H** relative to **Eu.1** in water likely reflects differences in first‐sphere hydration and ligand shielding; the glutamic‐acid–derived pendant arm in **Eu.CO_2_H** may provide more effective steric protection of the Eu(III) ion than the acetate arm in **Eu.1**, reducing access of quenching O–H oscillators. For each Eu(III) complex, the lifetimes increased slightly in deuterated relative to non‐deuterated solvent, indicating that no solvent molecules are directly coordinated to the Eu(III) centre (*q* = 0) [53], as expected for nine‐coordinate complexes in which the pendant quinoline arm binds in a bidentate manner and blocks the axial coordination site. Importantly, the long emission lifetime of **Eu.Chol** (0.71 ms) allows short‐lived cellular autofluorescence to be gated out while still capturing ONOO^−^ dynamics, whose lifetime in biological media is significantly longer (10–20 ms) [21] highlighting its potential for time‐resolved measurements in living cells.

### 2.4 Mechanism of Sensing ONOO^−^

The ability of the novel complex **Eu.Chol** to detect peroxynitrite was evaluated in 80:20 PBS/MeOH. Addition of 1 equivalent of ONOO^−^ to **Eu.Chol** (25 µM) caused rapid quenching of Eu(III) luminescence, with ∼80% loss within the first minute and ∼90% within 5 min (Figures 3 and S9), consistent with fast oxidative cleavage of the phenylboronic acid group and in situ formation of the corresponding 8‐hydroxyquinoline complex. Direct evidence for this transformation was provided by high‐resolution mass spectrometry (Figure 4), where we observe complete consumption of **Eu.Chol** (*m*/*z* = 1227.58) and formation of the corresponding Eu(III) product containing the 8‐hydroxyquinoline moiety (*m*/*z* = 1115.51). The observed isotopic distributions matched the theoretical patterns closely (Figure S10).

**FIGURE 3:**
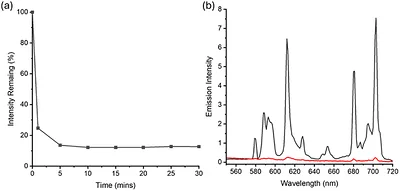
(a) Time‐dependent decrease in integrated Eu(III) emission (550–720 nm) of **Eu.Chol** (25 µM) upon treatment with 1 equivalent of ONOO^−^ over 30 min; (b) emission spectra of **Eu.Chol** (25 µM) before (black) and 30 min post (red) exposure to 1 equivalent of ONOO^−^. *λ*_exc_ 322 nm, 80:20 100 mM PBS/MeOH, pH 7.4, 295 K.

**FIGURE 4:**
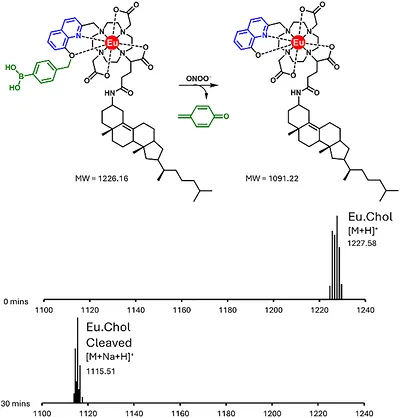
High‐resolution mass spectrometry (HR‐MS) data of **Eu.Chol** evidencing the cleavage of the phenylboronic acid moiety upon treatment with 1 equivalent of ONOO^−^, measured in methanol.

The absorption spectra also supported the reaction, with the broad band centred at 332 nm decreasing upon ONOO^−^ treatment and a strong peak emerging at 270 nm (Figure S16), which can be tentatively assigned to the π–π* transition of the resulting Eu(III)‐quinolinate complex, formed upon oxidative cleavage of the benzylboronic acid group. Collectively, this data supports a mechanism in which ONOO^−^ rapidly oxidises the phenylboronic acid group, triggering fast self‐immolation and formation of the 8‐hydroxyquinoline complex (Figure 4). This generates a dominant non‐radiative decay pathway for the Eu(III) excited state, likely *via* a charge‐transfer (or proton transfer) process in which the hydroxyl lone pair of the 8‐hydroxyquinoline, positioned near the Eu(III) centre, efficiently quenches luminescence. This sensing mechanism provides a rapid and sensitive readout for ONOO^−^ through selective quenching of Eu(III) emission.

As ONOO^−^ is highly reactive, it is commonly synthesized, stored, and delivered in alkaline solution (typically 0.3 M NaOH) to maintain stability. However, we considered that even small additions of concentrated NaOH could alter the pH of buffered samples and contribute to the observed changes in Eu(III) emission. Additional details concerning the NaOH‐induced changes in **Eu.Chol** emission are provided in the ESI (Figures S12–14). Control experiments revealed that elevated NaOH concentrations partially reduce **Eu.Chol** emission (Figure S12). To minimize this effect, we evaluated the impact of NaOH concentration on **Eu.Chol** emission and identified substantially lower basicity conditions that reduce this interference. Therefore, alternative ONOO^−^ preparation methods employing lower NaOH concentrations (0.1–0 M post‐synthesis dilution; see Supporting Information pages S3 and S4) were developed and enabled effective ONOO^−^ formation with only minimal impact on probe emission (Figure S15). Method 2c was chosen for the preparation of ONOO^−^ for the subsequent selectivity studies because it enabled delivery of equimolar ONOO^−^ with no measurable change in pH of the probe sample, yet **Eu.Chol** still exhibited rapid and substantial quenching, confirming that ONOO^−^ is the dominant contributor to the observed emission response.

## 3 RONS Selectivity Studies

The time‐dependent response of **Eu.Chol** toward ONOO^−^ was assessed in 80:20 PBS/MeOH (Figure 5a). To ensure all analytes were screened under the same conditions (pH 7.4, 295 K), Method 2c of ONOO^−^ preparation was used (Supporting Information pages S3 and S4), as it does not induce a measurable pH change. In this mixture, **Eu.Chol** responded rapidly to ONOO^−^, with 76% quenching of Eu(III)‐centred luminescence within 1 min after addition of 1 equivalent, consistent with fast cleavage of the phenylboronic acid group.

**FIGURE 5:**
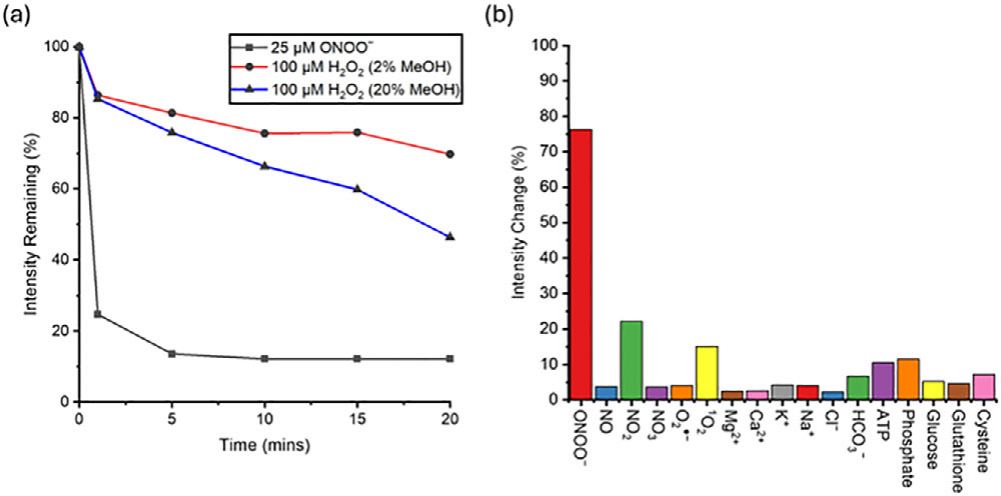
(a) The integrated Eu(III) emission response (550–720 nm) of **Eu.Chol** (25 µM) toward ONOO^−^ (prepared *via* method 2c, see Supporting Information pages S3 and S4) (25 µM, 80:20 100 mM PBS/MeOH) and H_2_O_2_ in fourfold excess (100 µM), measured in 80:20 100 mM PBS/MeOH and 98:2 100 mM PBS/MeOH over 20 min (b) Selective emission response of **Eu.Chol** (550–720 nm) (25 µM) toward 1 equivalent of ONOO^−^ (25 µM), compared with fourfold excess of other RONS: 100 µM NO, NO_2_^−^, NO_3_^−^, O_2_^•^^−^, ^1^O_2_; and biological analytes: 1 mM Mg^2^^+^, Ca^2^^+^, K^+^, Na^+^, Cl^−^, HCO_3_^−^, ATP, phosphate, glucose, glutathione and cysteine, measured after 1 min incubation. *λ*_exc_ 322 nm, 80:20 100 mM PBS/MeOH, pH 7.4, 295 K.

In contrast, exposure to a fourfold excess of H_2_O_2_ (100 µM) resulted in only approximately 15% emission decrease after 1 min, although prolonged exposure (20 min) led to approximately 50% quenching. To assess the effect of methanol on the H_2_O_2_ response, its proportion was reduced from 20% to 2% (98:2 PBS/MeOH), resulting in a slower reaction with only ∼25% emission quenching after 20 min. Reducing the MeOH content from 20% to 2% does not alter the immediate response toward ONOO^−^, which results in ∼80% emission quenching within 1 min (Figure S11). These results demonstrate that **Eu.Chol** can selectively detect ONOO^−^ within relevant timescales, and with minimal interference from H_2_O_2_.

The selectivity of **Eu.Chol** for ONOO^−^ was further demonstrated by incubating the probe with a range of reactive oxygen and nitrogen species in fourfold excess (100 µM NO, NO_2_^−^, NO_3_^−^, O_2_^•^^−^, ^1^O_2_), as well as common biological cations (1 mM Mg^2^^+^, Ca^2^^+^, K^+^, Na^+^), anions (1 mM Cl^−^, HCO_3_^−^, ATP), and biomolecules (1 mM glucose, glutathione, cysteine) (Figures 5b, S19 and S20). Each RONS was prepared according to literature procedures (see Supporting Information) and added to **Eu.Chol** (25 µM). The emission intensity was measured after 1 min and 20 min of incubation. After 1 min, nitrite (NO_2_^−^), superoxide (O_2_^•^^−^), and singlet oxygen (^1^O_2_) caused minor 10%–20% decreases in emission, reaching 20%–40% after 20 min. After 20‐min incubation with O_2_•^−^, a 40% decrease in **Eu.Chol** emission was observed, consistent with the gradual disproportionation of superoxide in aqueous solution and concomitant formation of H_2_O_2_, which is known to interact slowly with the Eu(III) probe (Figure 5a). In contrast, **Eu.Chol** exhibited a much larger and immediate emission decrease in response to ONOO^−^ at fourfold lower concentration, demonstrating that its high selectivity for ONOO^−^ is retained. The small change in emission observed with NO_3_^−^ after 20 min may have arisen from a weak interaction with **Eu.Chol**, potentially affecting the second coordination sphere upon equilibration. However, this effect is not considered significant, as the emission change is less than 20% after 20 min and is negligible within the first minute. In contrast, the response to ONOO^−^ is essentially instantaneous, clearly distinguishing it from the delayed and minor effect observed with NO_3_^−^. Pleasingly, **Eu.Chol** emission was largely unaffected (<10% change) by a broad range of biologically relevant cations, anions, and biomolecules. The most notable interference was from the reactive species hypochlorite (OCl^−^), where addition of 50 µM led to an approximate 60% increase in emission intensity (Figure S17b,d) after 20 min. However, intracellular OCl^−^ levels are estimated to be below 1 µM [23], and it reacts rapidly with endogenous thioethers and amines (Met: 3.8 × 10^7^ M^−^^1^ s^−^^1^; His: 1.0 × 10^5^ M^−^^1^ s^−^^1^) [57], making it unlikely to compete with **Eu.Chol** in cellular environments. We note, however, that interference from OCl^−^ may occur under certain nonphysiological conditions.

## 4 Sensitivity Studies

**Eu.Chol** exhibited high sensitivity toward ONOO^−^, with emission intensity decreasing linearly upon each 1 µM addition and reaching ∼90% quenching at 10 µM (Figures 6a and S21). Notably, **Eu.Chol** also showed slightly enhanced sensitivity in the nanomolar range compared with the previously reported **Eu.1**: after ten incremental additions of 100 nM ONOO^−^, emission decreased by ∼55% for **Eu.Chol** versus ∼40% for **Eu.1** (Figures 6b and S22). In contrast, the anionic complex **Eu.CO_2_H** displayed markedly weaker responses under the same conditions, essentially losing nanomolar sensitivity. Incremental addition of ONOO^−^ up to 10 µM led to only ∼30% emission quenching (Figure S23), and interestingly, the first ONOO^−^ addition caused a ∼20% increase in emission. The small initial increase in emission may arise from subtle perturbation of the amphiphilic probe's organisation in aqueous buffer, producing more discrete, separated species with minimal self‐quenching. The subsequent gradual emission decrease, with low sensitivity, is attributed to electrostatic repulsion between the peripheral carboxylate of **Eu.CO_2_H** and ONOO^−^. The restoration of nanomolar sensitivity in **Eu.Chol** (Figure 6b) upon coupling the cholesterol unit to the carboxylic acid confirms that an overall neutral complex is required for highly sensitive and selective ONOO^−^ detection.

**FIGURE 6:**
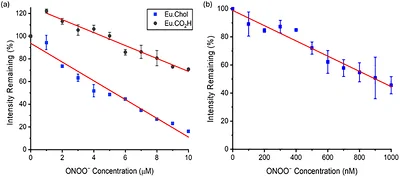
The integrated Eu(III) emission (550–720 nm) ± standard deviation following 5 min incubation with ONOO^−^. (a) Comparison of **Eu.Chol** and **Eu.CO_2_H** (25 µM) in the micromolar ONOO^−^ range (1–10 µM, 1 µM increments), highlighting the superior sensitivity of **Eu.Chol**. (b) Nanomolar ONOO^−^ sensitivity of **Eu.Chol** (100–1000 nM, 100 nM increments). *λ*_exc_ 322 nm, 80:20 100 mM PBS/MeOH, pH 7.4, 295 K.

## 5 Embedding **Eu.Chol** in Liposome Membranes

After demonstrating high selectivity, sensitivity, and a rapid response to ONOO^−^, we evaluated **Eu.Chol** in phospholipid membranes using model liposomes to confirm both its incorporation into the bilayer and its ability to detect ONOO^−^ in a membrane environment. Liposomes were prepared from 2‐oleoyl‐1‐palmitoyl‐*sn*‐glycero‐3‐phosphocholine (POPC, >99% purity) at a final lipid concentration of 0.4 mM in either HEPES (100 mM NaCl, 10 mM HEPES, pH 7.0) or PBS (100 mM PBS, pH 7.5) buffers. The emission intensity of **Eu.Chol** in PBS (4 µM) was nearly identical to that observed when it was added to liposomes (0.4 mM POPC), indicating that the liposomal environment does not perturb its emission. However, upon size exclusion chromatography (SEC) to remove unincorporated probe, a significant decrease in emission intensity after SEC was observed (Figure S24a).

Alternatively, the **Eu.Chol** stock solution was added to the lipid solution prior to solvent evaporation and liposome formation *via* hydration and extrusion through a 200 nm polycarbonate membrane. These liposomes with **Eu.Chol** pre‐incorporated exhibited an emission intensity comparable to that of **Eu.Chol** in solution, and the emission remained unchanged before and after SEC (Figure S24). These results indicate that **Eu.Chol** effectively incorporates into lipid membranes when included during liposome formation, while its post‐insertion into preformed membranes is limited. Subsequent studies on the emission quenching focused exclusively on LUVs with **Eu.Chol** prepared *via* pre‐incorporation.

The response of membrane‐embedded **Eu.Chol** toward ONOO^−^ was monitored using time‐resolved luminescence to selectively measure Eu(III) emission and remove short‐lived background fluorescence from the sample. Quartz cuvettes containing 3.0 mL of LUV suspension (0.4 mM POPC, 1 mol% **Eu.Chol**) were excited at 322 nm, and the emission intensity at 615 nm was monitored over one hour using a time‐gated acquisition. Upon cumulative addition of ONOO^−^ to **Eu.Chol** pre‐incorporated in LUVs, a gradual decrease in emission intensity was observed, reaching approximately half of the maximum reduction in emission (Figures 7a and S26). This partial loss of emission arises from the asymmetric distribution of **Eu.Chol** within the lipid bilayer: approximately half of the probe molecules reside in the outer leaflet, where they react rapidly with ONOO^−^ to form the non‐emissive product (Figure 7c,i–ii). However, access of ONOO^−^ to the inner leaflet is strongly restricted, not only by its negative charge but also by its very short lifetime in aqueous media (on the order of ∼10 ms), which limits diffusion across the membrane before decomposition (Figure 7c, iii).

**FIGURE 7:**
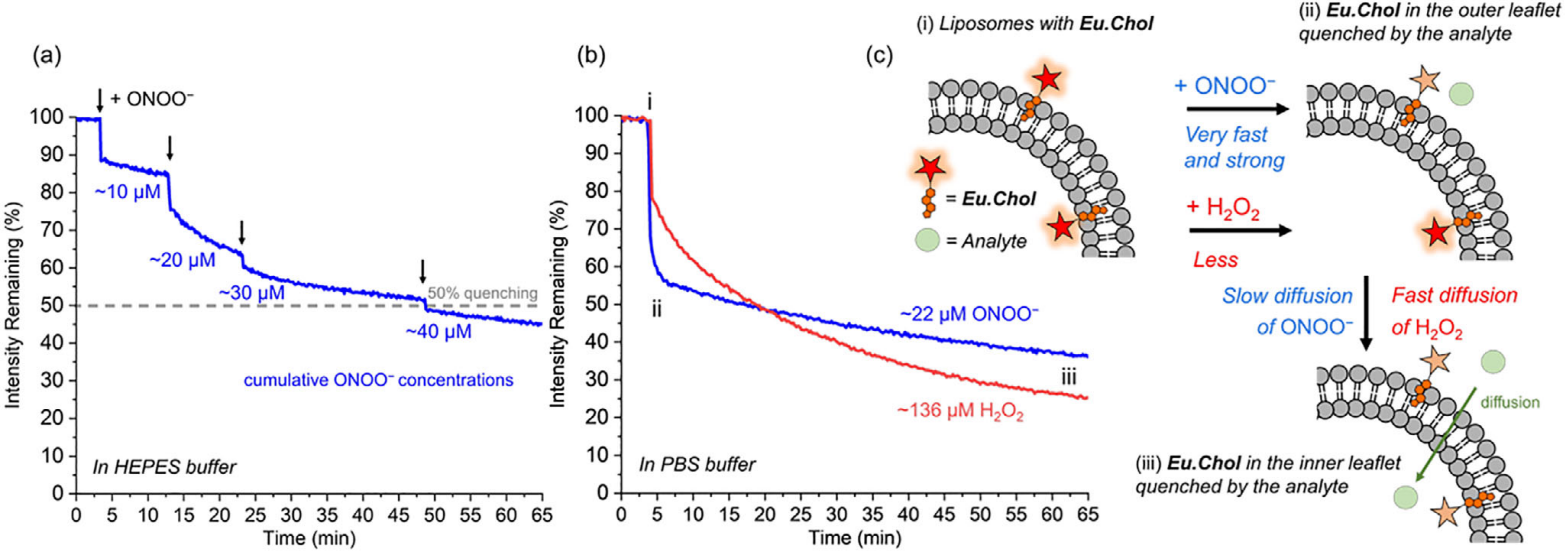
(a) Time‐resolved luminescence response (615 nm) of pre‐incorporated **Eu.Chol** (1 mol%, 4 µM) in POPC liposomes in HEPES buffer upon four subsequent additions of 10 µM ONOO^−^. Each ONOO^−^ pulse caused a rapid decrease in emission intensity, reaching a maximum reduction of ∼55%, consistent with the reaction of **Eu.Chol** is predominantly in the outer leaflet of the lipid bilayer. (b) Comparison of the time‐resolved luminescence response (615 nm) of pre‐incorporated **Eu.Chol** (1 mol%, 4 µM) in POPC liposomes in PBS buffer upon additions of ONOO^−^ (22 µM) or H_2_O_2_ (136 µM), showing a larger initial decrease upon addition of ONOO^−^, but a more significant decrease over time upon addition of H_2_O_2_. The final pH in the studies reached approximately 11. (c) Schematic representation of **Eu.Chol** in lipid membranes: (i) **Eu.Chol** pre‐incorporated into the liposome bilayer; (ii) reaction of ONOO^−^ or H_2_O_2_ with **Eu.Chol** in the outer leaflet leading to quenching; (iii) diffusion of analytes across the membrane enabling reaction with inner‐leaflet **Eu.Chol**, leading to complete emission loss.

In contrast, a sixfold larger concentration of H_2_O_2_ compared to ONOO^−^ is necessary to reach a similar overall decrease in emission intensity (Figure 7b). However, owing to its neutral character and longer lifetime compared to ONOO^−^ (seconds to hours under these conditions), H_2_O_2_ readily diffused across the lipid bilayer and affected **Eu.Chol** in both membrane leaflets (Figure 7c, iii). Importantly, the selective response of **Eu.Chol** toward ONOO^−^ over H_2_O_2_ enables these fundamental differences in membrane accessibility and lifetime to be directly observed within a lipid bilayer. This provides experimental evidence that externally generated ONOO^−^ is likely to act predominantly at the membrane or extracellular interface, whereas H_2_O_2_ is likely to penetrate lipid membranes and reach intracellular targets.

## 6 Flow Cytometry Application of **Eu.Chol**

Next, we evaluated **Eu.Chol** as a probe for monitoring cellular ONOO^−^ uptake in Jurkat leukemia suspension cells. An optimal concentration (50 µM) of **Eu.Chol** was predetermined in pilot experiments to ensure preservation of metabolic activity by the absence of toxicity at 24‐h post‐staining (Figure 8a). In addition, cells were exposed to varying concentrations of H_2_O_2_ or ONOO^−^ to identify suitable experimental exposure levels for H_2_O_2_ (100 µM) and ONOO^−^ (100 µM) using flow cytometry by identifying cell viability greater than 75% (IC_25_), 24‐h post‐exposure to the oxidants (Figure 8b). Next, the cells were stained with 50 µM **Eu.Chol**, incubated with nontoxic oxidant concentration (10–100 µM) for 60 min, and the probe emission was investigated using microplate reading and flow cytometry (Figure 8c).

**FIGURE 8:**
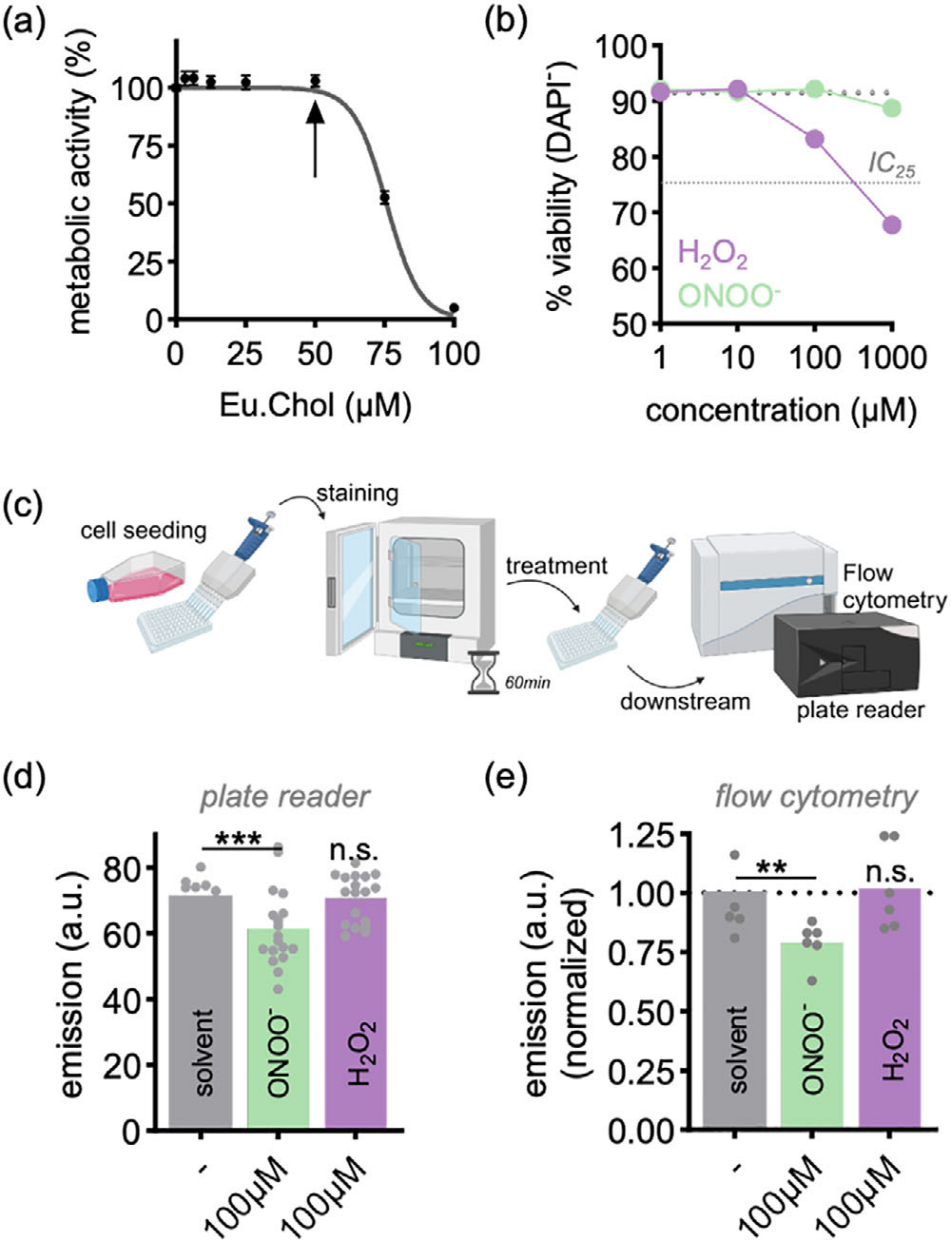
**Eu.Chol** studies in Jurkat cells. (a) Screening of **Eu.Chol** concentration against metabolic activity, indicating nontoxic concentration (arrow) used for subsequent experiments. (b) Titration of cells with increasing H_2_O_2_ and ONOO^−^ concentrations to determine reasonable treatment concentrations for subsequent experiments above 75% viability (IC_25_). (c) Schematic of the method used. (d) Selectivity screening of **Eu.Chol**‐stained cells against ONOO^−^and H_2_O_2_ using the microplate reader following excitation at 322 nm, with emission collected in time‐resolved mode using a 675/30 nm bandpass emission filter and an integration window of 60–400 µs. Bars represent normalised integrated Eu(III) emission intensity, with overlaid points indicating individual replicate measurements. (e) flow cytometry analysis of **Eu.Chol**‐stained cells showing differences in emission intensity following treatment with H_2_O_2_ versus ONOO^−^ for 10 min, measured in steady‐state mode using a 675/30 nm bandpass emission filter; bars expressed as integrated and normalised emission intensity, with overlaid points indicating individual replicate measurements.

Evaluation of the time‐resolved emission intensity of **Eu.Chol** using a plate reader indicated a rapid accumulation of the probe at or within cells, and incubation with 100 µM ONOO^−^ caused a significant, approximately 20% decrease in emission intensity. In contrast, 100 µM H_2_O_2_ caused no significant (n.s.) change in emission, confirming the selectivity of **Eu.Chol** (Figure 8d). Similar observations were made using flow cytometry, where **Eu.Chol** accumulation was also observed. Representative flow cytometry emission intensity distributions and histogram overlays for untreated and ONOO^−^‐treated cells are shown in Figure S27. Upon exposure to ONOO^−^, a consistent reduction in emission was observed (using a 675/30 nm bandpass filter) following excitation at 322 nm, shown as normalised scores acquired in steady‐state mode (Figure 8e). Again, emission was not significantly changed upon exposure to H_2_O_2_.

These observations align with our liposome studies, where the maximum response of **Eu.Chol** to ONOO^−^ is approximately a 50% decrease, reflecting the distribution of the probe across the inner and outer leaflets of the bilayer. Considering the short lifetime, high reactivity, and negative charge of ONOO^−^, the 15%–30% decrease observed in cells likely reflects probe exposure at the cell interface, consistent with limited penetration of ONOO^−^ across the membrane under these conditions.

Taken together, these experiments provide proof‐of‐principle that **Eu.Chol** can serve as a selective cellular reporter for ONOO^−^ dynamics. Knowing which reactive species penetrate cells, and to what extent, is critical for understanding the therapeutic effects of CAP treatment and other RONS‐based therapies. Selective probes such as **Eu.Chol** can uniquely reveal these dynamics at the membrane and within living cells.

## 7 Conclusion

We have developed a novel Eu(III) probe **Eu.Chol** for membrane‐targeted, selective peroxynitrite detection with nanomolar sensitivity. The probe is derived from the **Eu.CO_2_H** scaffold and incorporates a cholesterol unit to enable targeted membrane localisation without compromising photophysical properties, allowing direct monitoring of ONOO^−^ at the cellular interface *via* flow cytometry and plate‐reader analysis.

Liposome studies confirmed incorporation of **Eu.Chol** into lipid bilayers and revealed that cumulative ONOO^−^ addition produces a maximal emission decrease of approximately 50%, reflecting the asymmetric distribution of the probe between inner and outer leaflets and minimal ONOO^−^ transport across the membrane. **Eu.Chol** accumulated in Jurkat cells, and ONOO^−^ selectively quenched emission while H_2_O_2_ had little effect, highlighting the probe's high selectivity and ability to report real‐time RONS dynamics. The 15–30% emission decrease aligns with restricted ONOO^−^ access at the cellular membrane, as demonstrated in liposome studies. Together, these observations indicate that externally applied ONOO^−^ largely reacts at the membrane surface and shows minimal transport across intact lipid bilayers.

The modular synthetic approach presented here allows future development of probes integrating ratiometric calibration with sub‐cellular targeting for quantitative, spatially resolved monitoring of RONS in complex chemical and biological systems. Such selective probes provide a means to determine which RONS can access and react at the cell surface, information that is crucial for defining how oxidative signals are initiated. This has direct implications for therapies such as CAP, where the identity of membrane‐accessible species underpins both mechanism and therapeutic optimisation in these emerging plasma‐based treatments.

## Conflicts of Interest

The authors declare no conflicts of interest.

## Supporting information



The authors have cited additional references within the Supporting Information [58, 59, 60]. Supporting information for this article is given via a link at the end of the document. This includes experimental procedures, synthesis and characterization of compounds and complexes, photophysical analyses and liposome studies.
**Supporting File**: chem71463‐sup‐0001‐SuppMat.docx.

## Data Availability

The data that support the findings of this study are available in the supplementary material of this article.
