# Supplementary material for: Cellular Membrane‐Targeted Europium(III) Probe for Selective and Time‐Resolved Detection of Peroxynitrite
Source: Chemistry. 2026 Jul 27;32(40):e71463. doi: 10.1002/chem.71463 (PMC13716002; doi:10.1002/chem.71463)
Supplement: Supplementary file 1 [file CHEM-32-e71463-s001.docx]

*Electronic Supplementary Information (ESI)*

**Cellular Membrane-Targeted Europium(III) Probe for Selective and Time-Resolved Detection of Peroxynitrite**

Jamie Webb^a†^, Rebecca Tiffany^a†^, Karolis Norvaisa^b^, Hennie Valkenier^b^, Lea Miebach^c,d^, Sander Bekeschus^c,e^, and Stephen J. Butler*^a^

*^a^ Department of Chemistry, Loughborough University, Epinal Way, Loughborough, LE11 3TU, UK*

*^b^ Engineering of Molecular NanoSystems, Ecole polytechnique de Bruxelles, Université Libre de Bruxelles (ULB), Avenue F.D. Roosevelt 50, CP165/64, 1050 Brussels, Belgium*

*^c^ ZIK plasmatis, Leibniz Institute for Plasma Science and Technology (INP), Felix-Hausdorff-Str. 2, 17489 Greifswald, Germany*

*^d^ Department of Hematology and Oncology, Greifswald University Medical Center, Sauerbruchstr., 17475 Greifswald, Germany*

*^e^ Department of Dermatology, Venerology, and Allergology, Rostock University Medical Center, Strempelstr. 13, 18057 Rostock, Germany*

^†^ These authors contributed equally to this work.

**Contents**

1. Materials and methods	S2
2. Synthesis and characterisation	S8
3. Photophysical measurements	S23
4. Liposome assays	S31
5. References	S34
1. **Materials and Methods**

***General Considerations***

Reagent-grade chemicals, including anhydrous solvents, were purchased from Sigma Aldrich and Fluorochem and used without further purification.

**Nuclear Magnetic Resonance Spectroscopy**

¹H and ¹³C{¹H}  NMR spectra were recorded in the stated deuterated solvent on a JEOL–ECS– 400 spectrometer (¹H at 399.78 MHz, ¹³C{¹H}  at 100.53 MHz) and JEOL–ECS–500 spectrometer (¹H at 500.16 MHz, ¹³C{¹H}  at 125.77 MHz) at 293 K. Chemical shifts are expressed in ppm and are adjusted to the chemical shift of the residual NMR solvent resonances (CDCl₃: ¹H δ = 7.26 ppm, ¹³C δ = 77.16 ppm, CD₃OD: ¹H δ = 3.31 ppm, ¹³C δ = 49.00 ppm, or DMSO-d6: ¹H δ = 2.50 ppm, ¹³C δ = 39.52 ppm). The coupling constants are expressed in Hz.

**Mass Spectrometry**

Liquid Chromatography Electrospray Mass spectra were recorded on a Shimadzu LC–2020 system with controlled using LabSolutions, version 5.86 SP1 software. The system operates in positive or negative ion mode, with acetonitrile as the carrier solvent. The flow rate was maintained at 0.7 mL/min over a gradient of 5 to 95% acetonitrile in water (0.03% formic acid) for 10 minutes. High-resolution mass spectra were recorded using a Thermofisher Q–Exactive Orbitrap mass spectrometer.

**Column Chromatography**

Column chromatography was performed using flash silica gel 60 (particle size 40–63 microns) purchased from Apollo Scientific or using the CombiFlash NextGen 100® auto-column purchased from Teledyne. Thin layer chromatography (TLC) was performed on aluminium sheets.

**High Performance Liquid Chromatography**

Preparative RP–HPLC was performed using a Waters 2489 UV/Visible detector performed at 254 nm, and a Waters 1525 Binary HPLC pump controlled via the Waters Breeze 2 HPLC system software. Separation was achieved using a semi–preparative XBridge C18 (5 μm OBD 19 Å 100 mm) column at a flow rate maintained at 17 mL/min. A solvent system composed of either water (0.05% formic acid) / methanol (0.05% formic acid), water / methanol or water (25 mM NH₄HCO₃) / acetonitrile was used over the stated linear gradient (usually 0 to 100% organic solvent over 10 minutes). Analytical RP–HPLC was performed using a XBridge C18 (5 μm 4.6 Å 100 mm) column at a flow rate maintained at 2.0 mL/min using the stated gradient and solvents.

**pH Measurements**

Measurements of pH were carried out using a Jenway 3510 pH/mV meter with a Jenway combination electrode or a Jenway 3020 pH meter with an Aldrich glass combination pH electrode, both calibrated using buffer solutions of pH 4.00 ± 0.01, 7.00 ± 0.01 and 10.00 ± 0.01.

**Optical Spectroscopy**

Emission spectra were recorded on a SPEX Fluoromax luminescence spectrometer using dM3000 version 3.12 software. Emission spectra were obtained using either a 40 μL Hellma® Analytics quartz cuvette (Art no. 111–10–K–40) or a 1 mL Hellma® Analytics quartz cuvette (Art no. 101–10–K–40), with excitation at 322 nm and reading emission in the range 550 – 720 nm using an integration time of 0.5 seconds, increment of 1 nm and excitation and emission slits of 0.5 nm. UV/Visible absorbance spectra were measured using a Shimadzu UV–1800 UV– spectrophotometer.

***Determination of photophysical parameters***

Emission lifetime measurements were performed on the FluoroSENS instrument. Measurements were taken of 40 μL of 5 μM samples of Eu(III) complexes in 50 mM TRIS at pH 7.4. Measurements were obtained by indirect excitation of the Eu(III) ion via the quinoline antennae using a short pulse of light at 322 nm followed by monitoring the integrated intensity of the light emitted at 620 nm, with 500 data points collected over a 5 millisecond period. The decay curves were plotted in Origin Labs 2019 version 9.6.0.172, and fitted to the equation:

*I* = *A*_0_ + *A*_1_e^−^*^kt^*

where *I* is the intensity at time, *t*, following excitation, *A*_0_ is the intensity when decay has ceased, *A*₁ is the pre – exponential factor and *k* is the rate constant for the depopulation of the excited state.

The hydration state, *q*, of the Eu(III) complexes was determined using the modified Horrocks equation:^3^

*q*(Eu) = 1.2 (1/*τ*H_2_O − 1/*τ*D_2_O − 0.25 − 0.075*n*)

where *τ*_H₂O_ and *τ*_D₂O_ are the emission lifetime times in water and D₂O, respectively, and *n* is the number of carbonyl – bound amide NH groups (zero for the complexes herein).

**Preparation of stock solutions of reactive species**

OCl⁻ - A stock solution of hypochlorite (OCl⁻) was prepared from 5% active chlorine solution purchased from Sigma Aldrich in deionised deoxygenated water. The stock solution was used for subsequent dilutions, which were made fresh and used quickly to prevent degradation due to pH sensitivity.

ONOO⁻ - Stock solutions of peroxynitrite (ONOO⁻) were prepared fresh at the start of the day and reused in the same day. Three reagent solutions were prepared according to Method 1A:

Solution 1 – 0.7 M HCl, 0.6 M H₂O₂, 10 mL

Solution 2 – 0.6 M Sodium Nitrite, 10 mL

Solution 3 – 3.0 M NaOH solution, 5 mL

These solutions were simultaneously injected into an argon-flushed round bottom flask on ice. The flow rates were adjusted such that for every 1 mL of the 5 mL solution injected, 2 mL of each of the 10 mL solutions were added. All three solutions were dispensed continuously until depletion, ensuring all syringes were emptied at the same time. A clear, yellow solution indicated successful synthesis, and the round-bottom flask transferred to dry ice to freeze.

Synthesis of ONOO⁻ was confirmed by UV-VIS spectrometry showing a lack of nitrite presence. Dilutions were made with cold 0.3 M NaOH and prepared fresh prior to each experiment; the concentration was determined by defrosting a minimal amount of ONOO⁻, in a dark environment, ensuring it never defrosts more than an ice slurry and measuring the absorbance using UV-VIS (ε = 1670 ± 50 cm⁻¹M⁻¹ at 302 nm). Because the concentration of the stock solution varied between preparations, it was determined by UV–Vis spectroscopy for each batch to ensure accurate ONOO⁻ additions. A secondary working stock solution was then prepared, with the concentration adjusted depending on the experimental format (e.g., 1 μM titration, 100 nM titration, or single-addition experiments) to minimise addition volumes in each case. All solutions were kept on dry ice while in use and throughout the process light exposure was reduced to a minimum.

To assess the impact of NaOH concentration during the synthesis of ONOO⁻, five additional preparation methods were developed. These methods altered both the NaOH concentration used during synthesis and the subsequent NaOH concentration used for dilution (0.3 M, 0.1 M, or 0 M [water]):

**Method 1:**

Solution 1 – 0.7 M HCl, 0.6 M H₂O₂, 1 mL

Solution 2 – 0.6 M Sodium Nitrite, 1 mL

Solution 3 – 3.0 M NaOH solution, 0.5 mL

**A** – diluted in 0.3 M NaOH solution, as above.

**B** – diluted in 0.1 M NaOH solution.

**C** – diluted in water.

**Method 2:**

Solution 1 – 0.7 M HCl, 0.6 M H₂O₂, 1 mL

Solution 2 – 0.6 M Sodium Nitrite, 1 mL

Solution 3 – 1.5 M NaOH solution, 0.5 mL

**A** – diluted in 0.3 M NaOH solution.

**B** – diluted in 0.1 M NaOH solution

**C** – diluted in water.

The emission data of **Eu.Chol** corresponding to each ONOO⁻ preparation method are shown in Figures S12 and S15.

H₂O₂ - A stock solution of hydrogen peroxide (H_2_O_2_) was purchased from Sigma Aldrich (30% H₂O₂/H₂O, v/v%). The stock solution was used for subsequent dilutions into deionised deoxygenated water. Stocks were stored for future use.

O₂^•^⁻- Superoxide (O₂^•^⁻) was generated from KO₂. KO₂ and 18-crown-6 ether (2.5 equivalents) were dissolved in DMSO to afford a 0.25 M solution. The stock solution was used for subsequent dilutions.

¹O₂ - Singlet oxygen (¹O₂) was generated in situ from the photosensitisation of rose bengal. Rose bengal (100 μM) was added to [Eu.X] solutions (25 μM), and a full screen was conducted (scan every 2 minutes, over 30 minutes). No emission changes were observed. [Eu.X] + rose bengal solution was pumped through a photoreactor for 10 minutes, three times (λ_ex_ = 550 nm), and the emission intensity was analysed.

NO - Nitric oxide (NO) was prepared by dissolving diethylamine NONOate sodium salt (DEANO) in deionised deoxygenated water. The stock solution was used for subsequent dilutions.

NO₂⁻ - Nitrite (NO₂⁻) was prepared by dissolving sodium nitrite in deionised deoxygenated water. The stock solution was used for subsequent dilutions.

NO₃⁻ - Nitrate (NO₃⁻) was prepared by dissolving sodium nitrate in deionised deoxygenated water. The stock solution was used for subsequent dilutions.

PBS Stock (100 mM, 1 L) - To a solution (H₂O, 800 mL) of NaCl (80 g, 1.4 mol) in a 1L volumetric flask was added KCl (2.0 g, 27 mmol) and K₂HPO₄ (2.4 g, 18 mmol). The solution was stirred until all reagents fully dissolved. The pH was initially 6.68 and adjusted to 7.41 using 2 M NaOH. The solution was made up to 1 L with H₂O and stored in the fridge.

**General procedure for analysing time-dependent responses towards reactive species**

A known concentration stock solution of reactive species was prepared in water at pH 7.4. A known volume of each was added to 100 μL of the Eu(III) complex (25 μM, 80:20 100 mM PBS/MeOH, pH 7.4) and an emission spectrum was taken at 1 minute, then every 5 minutes for 30 minutes. Changes in intensity were compared with initial intensity. The aqueous buffer was not degassed prior to the addition of peroxynitrite, to maintain conditions consistent with the subsequent cell-based experiments, which were also performed under non-degassed conditions.

The percentage change compared to blank was calculated using:

$$\frac{(Sum of bands 550 - 720: Complex + RONS)}{(Sum of bands 550- 720: Complex only)}\times100=\% change$$

**General procedure for titration of peroxynitrite**

1 µL aliquots of ONOO⁻ were added to 200 µL **Eu.Chol** (25 µM) in 80:20 100 mM PBS/MeOH and the emission spectra recorded 5 mins after each addition. The ratio of the sum of all bands (550 – 720 nm) of each spectra compared to the complex alone was used to calculate the percentage change at each ONOO⁻ concentration.

**General procedure for the preparation of liposomes**

Stock solutions of 2-oleoyl-1-palmitoyl-sn-glycero-3-phosphocholine (POPC) (purity > 99%) were prepared in freshly deacidified chloroform and used for the preparation of the liposomes. Buffer solutions were prepared in water that was deionized with a Millipore filtration system:

HEPES buffer solution: NaCl (100 mM) and HEPES (10 mM) buffered at pH 7.

PBS buffer solution: NaCl (137 mM), KCl (2.7 mM), Na₂HPO₄ (10 mM) and KH₂PO₄ (1.8 mM) buffered at pH 7.5.

A lipid solution with POPC was prepared using volume calculated to obtain a final lipid concentration of 0.4 mM. The **Eu.Chol** probe stock solution 96 mM was made in MeOH, and the corresponding amount was used to make 1 mol% of probe to lipid ratio. For pre-incorporation studies, at this point, the **Eu.Chol** probe solution was added. The solvent was evaporated under a flow of nitrogen, and the resulting lipid film was dried under vacuum for at least 1 hour.

The lipid films were hydrated with 500 µL in their corresponding buffer solution (see the section above), sonicated for ca. 30 s, and then stirred for 1 hour at room temperature to create a heterogeneous mixture of vesicles. The suspension was subjected to 10 freeze-thawing cycles to generate unilamellar vesicles, diluted to 1 mL with the same salt solution previously employed for the hydration of the lipid film, and extruded 29 times through a polycarbonate membrane (200 nm pore size) at room temperature. For post-insertion studies, at this point, the **Eu.Chol** probe solution (to make 1 mol% probe to lipid ratio) was added. The large unilamellar vesicles (LUVs) suspension was eluted through a size exclusion column (Sephadex G-25) using the external salt solution as eluent to remove the external fluorescent probe. The resulting suspension was further diluted with the same salt solution to obtain the desired final lipids concentration of 0.4 mM, assuming no loss of lipids.

**Preparation of reactive species for liposome studies**

Peroxynitrite (ONOO⁻) stock solutions were freshly prepared each time prior to usage. Three solutions were made and injected at a similar flow rate into an argon-flushed round-bottom flask at ~20 mL/min on ice.

Solution 1 – 0.7 M HCl, 0.6 M H₂O₂, 1 mL

Solution 2 – 0.6 M Sodium Nitrite, 1 mL

Solution 3 – 3.0 M NaOH solution, 0.5 mL

All solutions were made up at the start of the day. Upon synthesis, the round-bottom flask was moved onto dry ice to freeze. The 100 µL of the stock solution was taken and diluted to 10 mL with cold 0.3 M NaOH. The concentration of formed ONOO⁻ was calculated using UV-VIS reading (ε = 1670 ± 50 cm⁻¹M⁻¹ at 302 nm).

Hydrogen peroxide (H₂O₂ ) was prepared from solution 1 by taking 20 µL and diluting to 5 mL with cold 0.3 M NaOH to make H₂O₂ (2.4 mM). This concentration corresponds directly to the H₂O₂ used in making ONOO⁻.

**Figure S1.** UV-vis reading and concentration calculation (ε = 1670 ± 50 cm⁻¹M⁻¹ at 302 nm) of formed ONOO⁻ showing the difference between ONOO⁻ production in different laboratories (a) ONOO⁻ formed in the Valkenier laboratory for liposome experiments; (b) ONOO⁻ formed in the Butler laboratory for the photophysical measurements.

**General procedure for time-gated emission studies of liposomes**

Quartz cuvettes containing 2.50 mL of LUV suspension with the probe **Eu.Chol** encapsulated were placed in the sample compartment of an Agilent Cary Eclipse fluorescence spectrometer equipped with a xenon flash lamp and a magnetic stirrer. Excitation and emission filters (ex.: 250 – 395 nm, em.: 430 – 1100 nm) were employed. The samples were excited at 322 nm (20 nm slits) and the emission intensity at 615 nm (5 nm slits) was monitored over 1 hour. A time-gate was applied (0.1 ms delay, acquisition time 2 ms, total decay time 3 ms). Four transport experiments were run in parallel. The final pH of the sample was approximately 11, following addition of the pulse of reactive species (150 µL of ONOO⁻ or H₂O₂ in 0.3 M NaOH).

**Cell culture**

Human T cell leukemia cells (Jurkat, CVCL_0065) were used in this study. Cells were cultured in Roswell Park Memorial Institute (RPMI) 1640 Medium supplemented with 10 % FBS (fetal bovine serum), 1% penicillin/streptomycin and 1 % glutamine. Cells were incubated at 37°C, 5% CO₂ and 95% humidity.

**Dose-finding of Eu.Chol for cellular experiments**

In order to identify an optimal concentration of **Eu.Chol** for subsequent experiments, cells were seeded at a density of 5 × 10⁴ cells per well in 90 µl RPMI without FBS in 96-well flat-bottom plates. Cells were exposed to **Eu.Chol** at different concentrations and incubated for 20 h. After 20h, metabolic activity of cells was evaluated using the resazurin assay. Briefly, 10 µL of resazurin reagent was added, followed by incubation under cell culture conditions for 4h. Viable cells metabolize non-fluorescent resazurin to fluorescent resorufin in a NADPH/H+-dependent reaction. Fluorescence intensity was measured at λ_ex_ 535 nm and λ_em_ 590 nm using a multimode plate reader (Infinite F200 Pro; Tecan, Switzerland).

**Dose-finding of hydrogen peroxide and peroxynitrite**

In pilot experiments, cells were exposed to different concentrations of H₂O₂ and ONOO⁻ to identify an optimal concentration for subsequent experiments. Cells were seeded at a density of 2 × 10₅ cells per well in 250 µL RPMI without FBS into 24-well flat-bottom plates. Immediately after, cells were exposed to different concentrations of the respective oxidant. After incubation for 4h, 250 µL RPMI without FBS was added to each well. After incubation for 24h, cells were harvested and stained with 1 µM DAPI (4′,6-diamidino-2-phenylindole; BioLegend, The Netherlands). After washing, cells were analysed using flow cytometry (CytoFLEX LX; Beckman-Coulter, Germany). Raw data analysis was performed using Kaluza 2.4 analysis software (Beckman-Coulter).

**Evaluation of Eu.Chol emission intensity in cells following exposure to peroxynitrite**

Cells were stained with 50 µM **Eu.Chol** in 5 mL 12 × 75 mm (FACS) tubes at a density of 3 × 10⁶ cells in 200 µL for 30 min on ice. After washing, cells were exposed to 10 µM and 100 µM of ONOO^–^ or 100 µM H₂O₂, respectively. Immediately after treatment, cells were transferred to a 96-well flat-bottom plate and analysed using a multimode plate reader (Infinite M200 Pro; Tecan), where emission intensities were recorded with excitation at 322 nm and emission collected at 660–690 nm in time-resolved fluorescence (TRF) mode using an integration window of 60–400 μs. Immediately thereafter, cells were analysed by flow cytometry (CytoFLEX LX; Beckman-Coulter) using the same excitation and emission wavelengths but in steady-state detection mode, as time-resolved acquisition was not available on this instrument. Raw data analysis was performed using Kaluza 2.4 software (Beckman-Coulter).

2. **Synthesis and Characterisation of Compounds**

**Synthesis of precursor complex Eu.Iodo**

**Scheme S1.** Synthesis of precursor complex **Eu.Iodo.**

2-Bromopentanedioic acid (**5**)

A solution of *L*–glutamic acid (10.0 g, 68 mmol) and sodium bromide (10.0 g, 97 mmol) in 2 M hydrobromic acid (50 mL) was cooled to –5 °C. Sodium nitrite (7.0 g, 101 mmol) was taken up in minimal water and added dropwise over 2 hours maintaining the temperature at –5 °C and the reaction allowed to warm to room temperature over a further 30 minutes. Conc. sulfuric acid (0.6 mL) was added and extracted with diethyl ether (5 x 100 mL). The organic phases were combined, backwashed with brine (2 x 50 mL) and dried over magnesium sulfate, solvent was removed under reduced pressure to give 2–bromopentanedioate **5** (5.2 g, 37%) as a yellow oil. ¹H NMR (500 MHz, CDCl₃) *δ* 4.31 (1H, dd, 3*J*_H–H_ = 8.5, 4*J*_H–H_ = 5.9 Hz, H2), 2.45–2.55 (2H, m, H4), 2.32–2.42 (2H, m, H3); ¹³C NMR (100 MHz, CDCl₃) *δ* 185.8 (C1), 180.6 (C5), 29.8 (C2), 27.8 (C4), 25.9 (C3); HR–MS ESI: 232.9421 [M+Na]⁺ requires [C₅H₉O₄BrNa]⁺ 232.9425; ν_max_/cm⁻¹ 2955 (CH), 1710 (C=O), 1153 (CO); Rf = 0.15 (Pet Ether/EtOAc 19:1 *v/v*). The data was in agreement with those reported previously.^57^

Di-*tert*-butyl 2-bromopentanedioate (**6**)

To a solution of 2-bromopentanedioic acid **5** (500.0 mg, 2.37 mmol) in dichloromethane (50 mL); a solution of *tert*–butyl 2,2,2–trichloroacetimidate (3.1 g, 14.21 mmol) in dichloromethane (20 mL) was added dropwise over one hour. Reaction mixture was stirred at room temperature for 3 hours before the solvent was removed to give the crude product, which was purified *via* column chromatography (silica gel, pet ether/EtOAc neat to 19:1, *v/v*) to give di–*tert*–butyl 2–bromopentanedioate **6** (444.0 mg, 58%) as a colourless oil. ¹H NMR (500 MHz, CDCl₃) δ 4.22 (1H, dd, 3*J_H-H_* = 8.5 Hz, 4*J_H-H_* = 5.9 Hz, H2), 2.48 – 2.34 (2H, m, H4), 2.33 – 2.23 (1H, m, H3_a_), 2.23 – 2.10 (1H, m, H3_b_), 1.46 (9H, s, H9), 1.43 (9H, s, H7); ¹³C NMR (100 MHz, CDCl₃) δ171.5(C5), 168.6 (C1), 82.6 (C8), 81.0 (C6), 47.0 (C2), 32.9 (C3), 30.0 (C4), 28.2 (C9), 27.9 (C7); HR–MS ESI 323.0607 [M+H]⁺ [C₁₃H₂₁O₄BrH]⁺ (requires 323.0852); Rf = 0.38 (pet ether/EtOAc 19:1 *v/v*). The data was in agreement with those reported previously.^57^

8-Hydroxyquinoline-2-carbaldehyde (**1**)

To a solution of commercially available 8–hydroxyquinoline (500.0 mg, 3.14 mmol) in 1,4-dioxane (7.5 mL), selenium dioxide (435.0 mg, 3.93 mmol) was added to a Schlenk round bottom in anhydrous conditions. The bright yellow solution was stirred for 19 hours at 80 °C and monitored hourly from 14 hours *via* TLC. The purple reaction mixture was filtered over celite® and washed with ethyl acetate (3 x 10 mL). The solvent was removed under reduced pressure. A dark orange solid was collected as the crude product, which was purified *via* column chromatography (Silica, 5–15% gradient EtOAc:Hex) to give the aldehyde product **1** as a yellow solid (289.5 mg, 54%). ¹H NMR (400 MHz, CDCl₃) *δ* 10.21 (s, 1H, H9), 8.31 (d, *J* = 8.5 Hz, 1H, H4), 8.05 (d, *J* = 8.5 Hz, 1H, H3), 7.62 (t, *J* = 8.0 Hz, 1H, H6), 7.42 (d, *J* = 8.0 Hz, 1H, H5), 7.28 (d, *J* = 8.0 Hz, 1H, H7); ¹³C NMR (100 MHz, CDCl₃) *δ* 192.9 (C9), 153.1 (C2), 150.2 (C8), 137.9 (C8’), 137.6 (C3), 131.1 (C6), 130.5 (C4’), 118.2 (C5), 118.1 (C4), 111.3 (C7); Rf = 0.35 (20% EtOAc/Hex). The data was in agreement with those reported previously.^34^

8-((4-Iodobenzyl)oxy)2-carbaldehyde (**2**)

To a solution of compound **1** (174.0 mg, 1.01 mmol) and potassium carbonate (214.0 mg, 1.54 mmol) in anhydrous acetonitrile (7.5 mL) was added 4–iodobenzyl bromide (366.0 mg, 1.05 mmol). The reaction mixture was stirred for 48 hours. The resulting clear solution (pale pink precipitate) was centrifuged at 120 rpm for 3 minutes. The solution was decanted, and solid pellet washed thrice with CH₂Cl₂ (3 x 15 mL). The solvent was evaporated under reduced pressure and the resulting residue partitioned between CH₂Cl₂ and saturated NaCl (aq). The aqueous phase was extracted and washed with CH₂Cl₂ (3 x 15 mL). The combined organic phases were dried over MgSO₄, filtered and concentrated under reduced pressure. The crude product was purified *via* column chromatography (silica gel; 20–60% EtOAc/Hex) to give **2** as a pale–yellow solid (264.0 mg, 68% yield). ¹H NMR (400 MHz, CDCl₃) *δ* 10.31 (s, 1H, H14) *δ* 8.29 (d, *J* = 8.49 Hz, 1H, H4), 8.08 (d, *J* = 8.48Hz, 1H, H6), 7.73 (d, *J* = 8.07 Hz, 2H, H3, H5), 7.67 (d, *J* = 8.02 Hz, 1H, H5), 7.54 (t, *J* = 8.01 Hz, 1H, H7), 7.48 (d, *J* = 8.0 Hz, 2H, H11), 7.30 (d, *J* = 8.0 Hz, 2H, H12), 7.11 (d, *J* = 7.99 Hz, 1H, H7), 5.30 (s, 2H, H9); ¹³C NMR (100 MHz, CDCl₃) δ 193.9 (C14), 154.9 (C2), 151.7 (C8), 140.29 (C8’), 137.9 (C12), 137.5 (C10), 138.0 (C11), 130.1 (C6), 129.0 (C4), 126.4 (C4’), 120.3 (C5), 118.1 (C3), 111.2 (C7), 93.6 (C13), 70.2 (C9); HR–MS ESI: 389.9913 [M+H]⁺; [C₁₇H₁₃INO₂]⁺ requires 389.9913; Rf = 0.20 (25% EtOAc/Hex). The data was in agreement with those reported previously.^34^

(8-((4-Iodobenzyl)oxy)quinolin-2-yl)methanol (**3**)

To a solution of aldehyde **2** (159.0 mg, 0.41 mmol) in methanol (20 mL) was added sodium borohydride (20.3 mg, 0.54 mmol). The orange reaction mixture was stirred at RT for 72 hours. The solvent was removed under reduced pressure and the resulting residue was partitioned between CH₂Cl₂ (20 mL) and saturated NaCl (aq) (10 mL). The aqueous layer was extracted with CH₂Cl₂ (3 x 20 mL), and organic layers were combined, dried over MgSO₄ and concentrated under reduced pressure to give the desired alcohol **3** as a white solid (138.7 mg, 87%). ¹H NMR (400 MHz, CDCl₃) *δ* 8.13 (d, *J* = 8.5 Hz, 1H, H4), 7.72 (d, *J* = 8.0 Hz, 2H, H13), 7.67 (d, *J* = 8.0 Hz, 1H, H5), 7.50 – 7.45 (m, 2H, H6), 7.42 (d, *J* = 8.0 Hz, 1H, H3), 7.29 (d, *J* = 8.0 Hz, 1H, H12), 7.15–13 (d, *J* = 8.0 Hz, 1H, H7), 5.34 (s, 2H, H10), 5.0 (s, 2H, H9), 4.99 (br, 1H, OH); HR–MS ESI: 392.0137 [M+H]⁺[C₁₇H₁₅INO₂]⁺ requires 392.0142; Rf = 0.35 (50% EtOAc/Hex). The data was in agreement with those reported previously.^34^ Note: for subsequent reactions carried out on larger scales (>200 mg **2**), the reaction mixture was cooled to 0 °C and NaBH_4_ was added slowly to prevent the formation of side products.

(8-((4-Iodobenzyl)oxy)quinolin-2-yl)methyl methanesulfonate (**3b**)

To a solution of compound **3** (200.0 mg, 0.52 mmol) and triethylamine (78.0 mg, 110 *μ*L, 0.76 mmol) in anhydrous DCM (10 mL) was added methane sulfonyl chloride (58.2 mg, 40.0 *μ*L, 0.51 mmol). The orange reaction mixture was stirred at RT for 3 hours. The solvent removed under reduced pressure and the residue partitioned between CH₂Cl₂ (30 mL) & saturated NaCl (aq) (10 mL). The aqueous layer was extracted with CH₂Cl₂ (3 x 20 mL) & organic layers combined, dried over MgSO₄, and concentrated under reduced pressure to give the mesylate ether **3b** as a white solid (247 mg, 100% crude). ¹H NMR (400 MHz, CD₃OD) *δ* 8.16 (d, *J* = 8.5 Hz, 1H, H3), 7.72 (d, *J* = 8.0 Hz, 2H, H14), 7.58 (d, *J* = 8.5 Hz, 1H, H4), 7.45 (t, *J* = 8.0 Hz, 1H, H5), 7.37 (d, *J* = 8.0 Hz, 1H, H6), 7.29 (d, *J* = 8.0 Hz, 1H, H13), 7.04 (d, *J* = 8.0 Hz, 1H, H7), 5.52 (s, 2H, H9), 4.03 (s, 3H, H10), 3.10 (s, 2H, H11); LR–MS ESI: 469.9 [M+H]⁺ [C₁₈H₁₇INO₄S]⁺ requires 469.9; Rf = 0.25 (50% EtOAc/Hex). The data was in agreement with those reported previously.^34^

Di-*tert*-butyl 2,2’-(4-((8-(4-iodobenzyl)oxy)quinoline-2-yl)methyl)-1,4,7,10-tetraazacyclododecane-1,7-triyl)diacetate (**4**)

To a solution of *tert–butyl* protected DO2A (145.0 mg, 0.57 mmol) and potassium carbonate (157.0 mg, 1.14 mmol) in anhydrous acetonitrile (20 mL) was added mesylate ester **3b** (243.0 mg crude). The pale–yellow solution was stirred at 60 °C for 2 hours and centrifuged at 1500 rpm for 3 minutes. The organic layer was removed, and pellet washed with CH₂Cl₂ (2 x 10 mL). The organic layers were combined, and the solvent was removed under reduced pressure. The crude was purified *via* column chromatography (silica gel; 9:1 *v/v*% CH₂Cl₂ /CH₃OH) to give **4** (112.0 mg, 29%) as a pale brown solid. ¹H NMR (400 MHz, CD₃OD); *δ* 8.02 (d, *J* = 8.2Hz, 1H, H4), 7.59 (d, *J* = 8.2Hz, 2H, H12), 7.36 (d, *J* = 8.2, 1H, H3), 7.28 (m, 2H, H5, H7), 7.13 (d, *J* = 8.2, 2H, H11), 6.95 (d,d, *J* = 9.0, 1H, H6), 5.18 (s, 2H, H14), 3.86 (s, 2H, H9), 3.09 – 2.68 (m, 20H, H_cyclen_), 1.41 (s, 18H, H22); ¹³C NMR (100 MHz, CD₃OD) *δ* 170.6 (C18), 156.9 (C4’), 153.9 (C10), 139.9 (C2), 137.8 (C12), 136.9 (C3), 136.5 (C13), 129.3 (C11), 128.5 (C8’), 126.7 (C7), 122.6 (C4), 120.3 (C5), 110.7 (C6), 93.6 (C8), 81.6 (C21), 70.3 (C9), 57.5 (C_cyclen_), 53.9 (C_cyclen_), 51.5 (C14), 50.7 (C19), 49.7 (C_cyclen_), 46.9 (C_cyclen_), 28.3 (C22); HR–MS ESI: 774.3087 [M+H]⁺ [C₄₃H₆₃IN₅O₇]⁺ requires 774.3091; Rf = 0.25 (5:95 CH₃OH/CH₂Cl₂). The data was in agreement with those reported previously.^58^

Di-*tert*-butyl 2-(4,10-bis(2-(tert-butoxy)-2-oxoethyl)-7-((8-((4-iodobenzyl)oxy)quinoline-2-yl)methyl)-1,4,7,10-tetraazacyclododecane-1-triyl)pentanedioate (**7**)

To a solution of protected ligand **4** (85.9 mg, 0.22 mmol) and potassium carbonate (59.5 mg, 0.43 mmol) in anhydrous acetonitrile (15 mL) was added bromine glutamic acid arm **6** (104.7 mg, 0.32 mmol) and potassium iodide (3.65 mg, cat. 10%). The pale–yellow solution was stirred at 60 °C for 48 hours (after 24 hours an additional equivalent of potassium carbonate, and bromine arm were added) and centrifuged at 1500 rpm for 3 minutes. The organic layer was removed, and pellet washed with CH₂Cl₂ (2 x 10 mL). The organic layers were combined, and the solvent was removed under reduced pressure. The crude was purified *via* column chromatography (silica gel; 100:1 – 10:1 *v/v*% CH₂Cl₂ /CH₃OH) to give **7** (70.6 mg, 63%) as a pale brown solid. ¹H NMR (400 MHz, CD₃OD) *δ* 8.04 (d, *J* = 8.5 Hz, 1H, H4), 7.62 (d, *J* = 8.2 Hz, 2H, H12), 7.57 (d, *J* = 8.5 Hz, 1H, H3), 7.31 (m, 2H, H5, H7), 7.17 (d, *J* = 8.2 Hz, 2H, H11), 6.95 (m, 1H, H6), 5.28 (m, 2H, H9), 3.64 – 1.82 (m , 26H, H_cyclen_, H14, H23–25), 1.45 – 1.30 (m, 36H, H22, H28, H31); ¹³C NMR (100 MHz, CD₃OD) δ 173.1(C29), 172.9 (C26), 172.3 (C18), 157.5 (C4’), 153.6 (C10), 137.7 (C2), 137.5 (C12), 136.8 (C3), 136.7 (C13), 128.7 (C11), 127.8(C8’), 126.6 (C7), 122.5 (C4), 120.2 (C5), 111.0 (C6), 93.2 (C8), 82.9 (C27), 82.5 (C30), 80.7 (C21), 70.3 (C9), 60.9 (C25), 60.5 (C24), 56.5 (C^cyclen^), 53.5 (C^cyclen^), 52.8 (C23), 51.5 (C14), 50.9 (C19), 50.0 (C^cyclen^), 47.4 (C^cyclen^), 28.3 (C22), 28.1 (C28), 27.7 (C31); HR–MS ESI: 1016.4605 [M+H]⁺ [C₅₀H₇₄IN₅O₉]⁺ requires 1016.4609; Rf = 0.25 (5:95 CH₃OH/CH₂Cl₂).

2-(4,10-Bis(2-(tert-butoxy)-2-oxoethyl)-7-((8-((4-iodobenzyl)oxy)quinoline-2-yl)methyl)-1,4,7,10-tetraazacyclododecane-1-yl)pentanedioic acid (**7a**)

To a solution of the protected macrocyclic ligand **7** (70.0 mg, 0.134 *μ*mol) in dichloromethane (1 mL), was added trifluoroacetic acid (2 mL) the resulting brown reaction mixture was stirred at room temperature for 24 hours and then the trifluoroacetic acid was co–evaporated with CH₂Cl₂ (5 × 10 mL). A brown oil was collected as the crude product, which was purified *via* preparative RP–HPLC [gradient: 0 − 100% acetonitrile in 25 mM NH₄HCO₃, over 15 minutes at 17 mL min⁻¹; Rt = 10.5 minutes] to give the deprotected ligand **7a** in its protonated form as a white solid (66.2 mg). NMR data could not be obtained due to lack of solubility of the deprotected ligand in a range of protic and non-protic organic solvents. HR–MS ESI: 792.2026 [M+H]⁺ [C₃₄H₄₂IN₅O₉]⁺ requires 792.2027.

**Eu.Iodo**

The deprotected ligand **8** (50.3 mg, 0.063 mmol) was dissolved in deionised water (5 mL) and the pH adjusted to pH 7.5 using 0.3 M NaOH solution. EuCl₃∙6H₂O (12.5 mg, 0.031 mmol) was added and the pH was readjusted to 7.5, before stirring the solution at 60 °C for 24 hours. The solvent was removed *via* freeze drying to give the desired Eu(III) complex **Eu.Iodo** (59.8 mg, 100%) as an orange solid, which was used in the next step without further purification. It was not possible to obtain NMR data for the iodo-functionalised Eu(III) complex **Eu.Iodo** due to its poor solubility in a range of protic and non-protic organic solvents. HR–MS ESI: 942.1076 [M+H]⁺ [C₂₉H₃₄IN₅O₉Eu]⁺ requires 942.1083. Reverse-phase HPLC analysis highlighted the lipophilicity of the complex, with elution occurring at 90% MeOH. The HPLC trace showed a single, well-defined peak, consistent with the presence of a single species in solution.

**Figure S2**: RP-HPLC trace of **Eu.Iodo** using a 10–100% MeOH/H₂O gradient over 18 minutes with a 7 minute MeOH hold, showing a single peak at 18.3 minutes (~91% MeOH).

**Synthesis of target complex Eu.Chol**

**Scheme S2.** Synthesis of target complex **Eu.Chol.**

(10*R*, 13*R*)-2-Azido-10,13-dimethyl-17-((*S*)-6-methylheptan-2-yl)-2,3,4,7,8,9,10,11,12,13,14,15,16,17-tetradecahyddro-1*H*-cyclopenta[a]phenathrene (**8**)

To a solution of cholesterol (2.00 g, 5.17 mmol) in anhydrous THF (20 mL) was added triphenylphosphine (1.63 g, 6.21 mmol) and diisopropyl azodicarboxylate (1.22 mL, 6.21 mmol), resulting in a yellow solution that was stirred for 10 minutes. Diphenylphosphoryl azide (1.34 mL, 6.21 mmol) was added and the reaction mixture stirred overnight at RT. The solvent was removed *via* rotary evaporation, and the crude material purified *via* column chromatography (silica gel; Hex) to give the azide **8** (1.23 g, 58%) as a light pink solid. ¹H NMR (400 MHz, CDCl₃) δ 5.37 (s, 1H), 3.30 – 3.13 (m, 1H), 2.26 (d, *J* = 7.9 Hz, 2H), 2.03 – 0.83 (m, 37H), 0.66 (s, 3H); FT-IR = ν_max_ = 2100, 1600 cm⁻¹; LR–MS ESI: 412.4 [M+H]⁺ [C₂₇H₄₆N₃]⁺ requires 412.4; Rf: 0.30 (Hex). The data were in agreement with those reported previously.^59^

(10*R*, 13*R*)-10,13-Dimethyl-17-((S)-6-methylheptan-2-yl)-2,3,4,7,8,9,10,11,12,13,14,15,16,17-tetradecahydro-1*H*-cyclopenta[a]phenathren-2-amine (**9**)

A solution of azide **8** (400 mg, 0.97 mmol) in anhydrous THF (2 mL) was prepared. Triphenylphosphine (2.55 g, 9.72 mmol) was dissolved in 5 mL anhydrous THF and added to the azide solution and the reaction mixture was stirred for 30 minutes at RT. Water (2 mL) was added and the mixture was stirred over night with a balloon to trap the released N₂ gas. The resulting solution was reduced *via* rotary evaporation and co-evaporated with toluene to remove excess water. The crude material was purified *via* column chromatography (silica gel; 5:1 v/v% CH₂Cl₂ /CH₃OH) to give **9** (153 mg, 35%) as a white solid. ¹H NMR (400 MHz, DMSO-d6) δ 5.49 (s, 1H, HA), 3.30 – 3.13 (m, 1H), 2.26 (d, *J =* 7.9 Hz, 2H), 2.03 – 0.83 (m, 37H), 0.66 (s, 3H); ¹³C NMR (100 MHz, DMSO-d6) δ 140.2, 123.0, 61.4, 57.1, 56.6, 50.4, 42.7, 40.0, 39.9, 38.3, 37.8, 36.63, 36.61, 36.4, 32.3, 32.2, 28.6, 28.3 (x2), 24.4, 24.1, 23.1, 23.0, 21.1, 19.8, 19.0, 12.2; FT-IR = ν_max_ = 1640 cm⁻¹ NH_2_, 1600 cm⁻¹ C=C; HR–MS ESI: 386.3782 [M+H]⁺ [C₂₇H₄₈N]⁺ requires 386.3781; Rf: 0.10 (5:95 CH₃OH/CH₂Cl₂). The data were in agreement with the reported literature.^59^

**Eu.Chol.Iodo**

**Eu.Iodo** (100 mg, 0.106 mmol) was dissolved in anhydrous DMF (2.1 mL, 0.05 M). HATU (121.2 mg, 0.318 mmol) and DIPEA (74 μL, 0.425 mmol) were added and stirred for two minutes with the solution turning from colourless to dark brown. Cholesterylamine **9** (45.00 mg, 0.117 mmol) was added and the mixture stirred under argon for 24 hours at RT. The resulting dark brown solution was co-evaporated with toluene under reduced pressure to afford a dark brown viscous oil. The crude product was dry loaded onto celite® and purified *via* RP–Combiflash® Auto-column [gradient: 0 − 100% methanol/water (0.05% formic acid), over 50 minutes at 13 mL min⁻¹] to give the Eu(III) complex **Eu.Chol.Iodo** as a white powder (19.8 mg, 14%). ¹H NMR: (400 MHz, CD₃OD) δ 53.2, 51.96, 49.93, 48.65, 42.10, 36.32, 32.08, 28.50, 26.68, 25.87, 24.26, 21.32, 21.00, 19.38, 17.78, 12.96, 12.04, 10.97, 10.39, -0.97, -1.56, -1.94, -2.31, -2.90, -3.76, -5.47, -6.39, -6.87, -7.03, -7.73, -8.53, -9.71, -10.08, -11.53, -13.62, -14.69, -15.17, -18.66, -20.37, -22.95, -23.48, -24.34, -25.57, -35.81; HR–MS ESI: 1331.4511 [M+Na]⁺ [C₆₁H₈₄EuIN₆NaO₈]⁺ requires 1331.4500; Rf = 0.3 (10:90 CH₃OH/CH₂Cl₂). The RP-HPLC chromatogram (Fig. S3) is consistent with the presence of two predominant diastereomers arising from chirality in both the glutamic acid-derived arm and the cholesterol unit, which together favour formation of two major isomeric Eu(III) complexes. The isomers were combined and taken forward for the synthesis of **Eu.Chol.**

**Figure S3.** RP-HPLC trace of **Eu.Chol.Iodo** in 100% MeOH (isocratic elution over 30 min) showing two overlapping peaks at 12.3 and 12.7 minutes. The complex eluted as two closely overlapping peaks, assigned to two diastereomers of **Eu.Chol.Iodo.** The fractions were collected as separately as possible and analysed by HRMS, which revealed the same molecular ion [M+Na]⁺ for both fractions.

**Figure S4.** ^1^H-NMR spectrum (400 MHz, CD₃OD) of **Eu.Chol.Iodo.**

**Eu.Chol**

**Eu.Chol.Iodo** (18.0 mg, 0.014 mmol) was dissolved in methanol (4 mL) and potassium acetate (4.9 mg, 0.061 mmol) and B₂Pin₂ (8.4 mg, 0.041 mmol) were added. The resulting mixture was degassed via freeze–pump–thaw (x3) prior to the addition of catalyst Pd(dppf)Cl₂ (0.92 mg, cat. 10%). The reaction mixture was stirred at 80 °C for 4 hours under nitrogen. The resulting dark brown solution was filtered over celite, and solvent removed under reduced pressure. The crude product was stirred for 1 hour in acidified methanol (0.05% formic acid) and then dry loaded onto celite® and purified *via* RP–Combiflash® Auto-column [gradient: 0 − 100% methanol/water (0.05% Formic Acid), over 50 minutes at 13 mL min⁻¹] to give the target complex **Eu.Chol** as a pale orange solid (7.1 mg, 42%). ¹H NMR: (400 MHz, CD₃OD); δ 53.57, 51.53, 50.27, 43.52, 37.11, 33.25, 30.52, 27.97, 26.95, 25.19, 22.97, 22.06, 20.53, 18.66, 14.17, 11.79, 11.16, -0.30, -0.70, -1.33, -1.55, -2.29, -3.08, -4.90, -6.95, -7.86, -8.82, -9.27, -10.86, -12.85, -13.13, -13.65, -14.55, -18.70, -19.54, -20.11, -23.00, -24.03, -29.40, -36.01; HR–MS ESI: 1249.5617 [M+Na]⁺ [C₆₁H₈₆EuN₆NaO₁₀]⁺ requires 1249.5603; Rf = 0.2 (10:90 CH₃OH/CH₂Cl₂). Photophysical analysis: λ_max_(H₂O) = 322nm; ε(H_2_O) = 5988 M⁻¹cm⁻¹; Φ(H₂O) = 3.3, Φ(MeOH) = 6.6; ν(MeOH) = 0.706 ms, ν(MeOH) = 0.780 ms; hydration state, q = -0.21.

**Figure S5.** ^1^H-NMR spectrum (400 MHz, CD₃OD) of **Eu.Chol**

***Synthesis of control complex Eu.CO₂H***

**Scheme S3.** Synthesis of complex **Eu.CO₂H** from **Eu.Iodo.**

**Eu.CO₂H**

The iodo-functionalised complex **Eu.Iodo** (108.0 mg, 0.124 mmol), was dissolved in methanol (20 mL) and potassium acetate (50.5 mg, 0.515 mmol) and B₂Pin₂ (87.0 mg, 0.348 mmol) was added. The mixture was degassed *via* freeze–pump–thaw (x3) prior to the addition of catalyst Pd(dppf)Cl₂ (9.0 mg, 0.0124 mmol). The reaction was stirred at 80 °C for 2.5 hours under nitrogen. The resulting dark brown solution was filtered over celite, and solvent removed under reduced pressure. The crude product was purified *via* preparative RP–HPLC [gradient: 10 − 100% methanol/water (0.05% formic acid), over 18 minutes at 19 mL min⁻¹; Rt = 10.5 minutes] to give the final complex **Eu.CO₂H** as a white solid (5.0 mg, 6%). HR–MS ESI: 860.2180 [M+2H]⁺ [C₃₄H₄₂BEuN₅O₁₁]⁺ requires 860.2181. Reverse-phase HPLC analysis highlighted the reduced lipophilicity of the complex compared with **Eu.Iodo**, with a single, well-defined peak eluting at % MeOH, consistent with introduction of the boronic acid group in place of the iodo substituent. Photophysical analysis: λ_max =_ 322 nm; ε(H₂O) _=_ 2879 M⁻¹cm⁻¹; Φ(H₂O) = 5.0%; Φ(MeOH) = 9.0%; τ(H₂O) = 0.63 ms, τ(D₂O) = 0.78 ms, hydration state, *q* = -0.02.

**Figure S6**: RP-HPLC trace of **Eu.CO₂H** using a 10–100% MeOH/H₂O gradient over 18 minutes with a 7 minute MeOH hold, showing a single peak at ~13 minutes (~64% MeOH).

3. **Photophysical Measurements**

**Figure S7.** Absorption spectra of (a) **Eu.1** measured in 100 mM PBS, pH 7.4, 295 K; (b) **Eu.CO₂H** (100 mM PBS, pH 7.4, 295 K) and (c) **Eu.Chol** (MeOH, 295 K)**.**

**Figure S8.** Emission spectra of (a) **Eu.1** measured in 100 mM PBS, pH 7.4, 295 K; (b) **Eu.CO₂H** (100 mM PBS, pH 7.4, 295 K) and (c) **Eu.Chol** (MeOH, 295 K), (λ_exc_ 322 nm).

**Figure S9.** **Eu.Chol** (25 µM) treated with 1 equivalent of ONOO⁻; (a) Change in emission intensity of the Eu emission (550 – 720 nm), Δ*J* = 2 band (605 – 630 nm) and Δ*J* = 4 band (675 – 720 nm) over 30 minutes (adapted from Figure 3 in main paper); (b) emission spectrum following exposure to ONOO⁻ (λ_exc_ 322 nm, 90:10 100 mM PBS/MeOH, pH 7.4, 295 K).

**Figure S10.** HR-MS data of **Eu.Chol** evidencing the cleavage of the phenylboronic acid moiety upon addition of ONOO⁻, measured in methanol (adapted from Figure 4 in main paper). For **Eu.Chol** in the absence of ONOO⁻, a lower-intensity signal centred at m/z = 1241.59 is also observed and assigned to the [M+H]⁺ ion of **Eu.Chol** bearing a methylated boronate (B(OH)(OMe)), arising from partial esterification under the ESI-MS conditions. For **Eu.Chol** in the presence of ONOO⁻, the [M+2H]^+^ ion is observed but at very low intensity (~3,000 counts) compared with the dominant [M+Na+H]^+^ signal (~80,000 counts).

**Figure S11.** **Eu.Chol** (25 µM) treated with 1 equivalent of ONOO⁻ in 2% and 20% MeOH with 100 mM PBS buffer (λ_exc_ 322 nm, pH 7.4, 295 K).

***Evaluation of NaOH interference and optimisation of ONOO⁻ preparation***

**Figure S12.** (a) **Eu.Chol** (25 µM, 200 μL total volume) treated with 5 μL 0.3, 0.1, 0.05, 0.025 and 0.01 M NaOH. (80:20 100 mM PBS:MeOH, λ_exc_ 322 nm, pH 7.4, 295 K).

**Figure S13.** (a) emission spectra and (b) absorption spectra of **Eu.Chol** (25 µM, 200 μL total volume) treated with 0.3M NaOH (red line) and pH adjusted back to pH 7 with HCl (blue line). Measured in 80:20 100 mM PBS:MeOH, λ_exc_ 322 nm, 295 K.

The impact of 0.3M NaOH on **Eu.Chol** was studied by emission and UV–Vis absorption spectroscopy (Fig. S13). Addition of 0.3M NaOH resulted in a significant decrease in Eu(III)-centred emission (Fig. S13a, red line) accompanied by an increase in ligand fluorescence in the 350–500 nm region, indicating disruption of Eu(III) sensitisation and suggesting at least partial Eu(III)–ligand decomplexation under strongly basic conditions. Upon subsequent readjustment of the pH to 7, Eu(III) emission partially recovered and ligand fluorescence decreased (blue line); however, the fluorescence-to-Eu(III) emission ratio did not return to that of the original probe (black line), indicating that the process is not fully reversible on the experimental timescale.

UV–Vis absorption spectroscopy (Fig. S13b) revealed a red shift of the lowest-energy absorption band of **Eu.Chol** in the presence of 0.3 M NaOH, which partially shifted back upon return to neutral pH, although the spectral shape was not fully restored. Notably, the absorption feature at 270 nm observed following treatment with ONOO⁻ (Fig. S16 below), assigned to the π–π* transition of the Eu(III)–quinolate species formed following oxidative cleavage of the benzylboronic acid group, was absent under NaOH conditions, indicating that hydroxide does not

induce oxidative cleavage of the boronic acid moiety.

**Figure S14.** HRMS data of **Eu.Chol** in the presence of 0.3 M NaOH in methanol. (a) Full mass spectrum showing a low signal-to-noise ratio and multiple m/z signals that could not be confidently assigned to discrete structures. (b) Expanded region highlighting the weak signal corresponding to the [M+Na]⁺ ion of **Eu.Chol,** which remained detectable under these conditions.

HRMS analysis was performed on **Eu.Chol** in the presence of 0.3 M NaOH (Fig. S14). Compared with the intact probe at neutral pH (Fig. S10), the spectra became substantially noisier, although a weak signal corresponding to **Eu.Chol** remained detectable. Multiple additional m/z features were observed but could not be confidently assigned to discrete structures, and no signal corresponding to the free ligand was detected. However, this does not exclude ligand formation, as the decomplexed ligand is expected to exist as a highly polar, polyanionic species under these conditions and may therefore ionise poorly by ESI-MS. Extensive optimisation of the analytical conditions, including variation of solvent composition, acidification strategy, source parameters, lock-mass calibration, and injection volume, did not significantly improve spectral clarity. Taken together, the UV and emission spectroscopic and HRMS data suggest that **Eu.Chol** undergoes partial demetallation accompanied by changes in the electronic structure of the quinoline antenna, rather than formation of a single well-defined hydroxide-derived product. These observations prompted the development of an ONOO⁻ preparation method employing substantially lower NaOH concentrations, thereby minimising hydroxide-induced perturbation of **Eu.Chol** and enabling assessment of ONOO⁻ reactivity under pH-controlled conditions (Fig. S15).

**Figure S15.** **Eu.Chol** (25 µM) treated with 1 equivalent of ONOO⁻ prepared *via* six methods detailed on pages S3 and S4. (80:20 100 mM PBS:MeOH, λ_exc_ 322 nm, pH 7.4, 295 K).

**Figure S16.** Absorption spectrum of **Eu.Chol** in MeOH before and after 1:1 µM treatment with ONOO⁻.

**Figure S17.** Emission response of 25 µM **Eu.Chol** (550 – 720 nm) to: (a) ONOO⁻ (25 µM) and H₂O₂ in 4-fold excess (100 µM) in 80:20 PBS/MeOH and 98:2 PBS/MeOH over 20 minutes (adapted from Figure 5 in main paper); (b) OCl⁻ (50 and 100 µM) over 20 minutes; (c) emission spectrum at 0 mins (grey) and 20 mins (red) following treatment with H_2_O_2_ (100 µM); (d) emission spectrum at 0 mins (grey) and 20 mins (red) following treatment with OCl⁻ (100 µM). (All except (a) measured in 80:20 100 mM PBS/MeOH, pH 7.4, 295 K, λ_exc_ 322 nm).

**Figure S18. Eu.Chol** (25 µM) treated with 100 μM H₂O₂ prepared under basic conditions (diluted in 0.3M NaOH) and normal conditions (diluted in water) in 20:80 MeOH:PBS buffer (λ_exc_ 322 nm, pH 7.4, 295 K).

**Figure S19.** Selective response of **Eu.Chol** (550 – 720 nm) (25 µM) towards ONOO⁻ (prepared *via* method 2c, see pages S3 and S4) (25 µM), over other RONS in 4-fold excess: 100 µM NO, NO₂⁻, NO₃⁻, O₂^•^⁻ and ¹O₂; after incubating for 1 minute and 20 minutes; as well as biological analytes: 1 mM Mg²⁺, Ca²⁺, K⁺, Na⁺, Cl⁻, HCO₃⁻, ATP, phosphate, glucose, glutathione and cysteine after incubating for 1 minute. (λ_exc_ 322 nm, 80:20 100 mM PBS/MeOH, pH 7.4, 295 K (adapted from Figure 5 in main paper).

**Figure S20.** Emission spectra highlighting the selective response of **Eu.Chol** (25 µM) towards ONOO⁻ (prepared *via* method 2c, see pages S3 and S4) (25 µM), over other RONS in 4-fold excess: 100 µM NO, NO₂⁻, NO₃⁻, O₂^•^⁻ and ¹O₂ after incubating for 20 minutes. (λ_exc_ 322 nm, 80:20 100 mM PBS/MeOH, pH 7.4, 295 K).

**Figure S21.** Micromolar sensitivity of **Eu.Chol** (25 µM) towards 1 – 10 µM ONOO⁻ added in 1 µM additions; (a) adapted from Figure 7a in main paper, the change in emission intensity (550 – 720 nm) following each addition; (b) emission spectra recorded 5 minutes post each addition. (λ_exc_ 322 nm, 80:20 100 mM PBS/MeOH, pH 7.4, 295 K).

**Figure S22.**  Nanomolar sensitivity of **Eu.Chol**. (a) Emission spectral changes of **Eu.Chol** (25 µM) upon addition of 100–1000 nM ONOO⁻ in 1 µL increments. Emission spectra were recorded 5 minutes after each addition; (b) Overall emission intensity change (550–720 nm) of **Eu.Chol** (25 µM) in the presence of 100–1000 nM ONOO⁻, adapted from Figure 7b in the main text (λ_exc_ = 322 nm, 80:20 100 mM PBS/MeOH, pH 7.4, 295 K); (c) Overall emission intensity change (550–720 nm) of **Eu.1** (25 µM) under identical ONOO⁻ concentrations, showing slightly lower sensitivity over this range compared with **Eu.Chol** (λ_exc_ = 321 nm, 100 mM PBS, pH 7.4, 295 K).

**Figure S23.** Reduced sensitivity of **Eu.CO_2_H** (25 µM) toward ONOO⁻. (a) Emission intensity (550–720 nm) changes upon incremental additions of 1–10 µM ONOO⁻ (1 µM steps). (b) Emission intensity (550–720 nm) changes upon incremental additions of 200–1000 nM ONOO⁻ (200 nM steps). (λ_exc_ = 322 nm, 80:20 100 mM PBS/MeOH, pH 7.4, 295 K).

4. **Liposome Assays**

**Figure S24.** Emission study of the **Eu.Chol** probe when incorporated into lipid bilayers. The emission spectra of the **Eu.Chol** probe (a) in HEPES buffer: in blue 4 µM of **Eu.Chol**, in black 4 µM (1 mol%) of **Eu.Chol** added before SEC, in red after SEC, and in green 4 µM (1 mol%) of **Eu.Chol** added during lipid film preparation (pre-incorporated); (b) in PBS buffer: in blue 4 µM of **Eu.Chol**, in black before SEC of 4 µM (1 mol%) of **Eu.Chol** pre-incorporated, and in red, after SEC.

We confirmed that the **Eu.Chol** (4 µM) probe exhibited a large, measurable decrease in emission intensity upon exposure to freshly prepared ONOO⁻ in both HEPES (Figure S25a) and PBS (Figure S25b) buffers in the lab where the liposomes were prepared. A substantially higher concentration of H₂O₂ was required to achieve a similar decrease in **Eu.Chol** emission, consistent with the selectivity for ONOO⁻ established above. Notably, a relatively large concentration of ONOO⁻ (~15 µM) was required to reduce **Eu.Chol** emission by more than 95%. This is attributed to differences in ONOO⁻ stock preparation between laboratories, reflected in the lower absorbance band characteristic of ONOO⁻ formation (Figure S1a), rather than a decrease in probe sensitivity. Overall, the selectivity of **Eu.Chol** for ONOO⁻ over H₂O₂ remains clear.

**Figure S25.** Control experiments for the ONOO⁻ preparation and **Eu.Chol** quenching in aqueous solution without liposomes. The emission spectra of 2-4 µM **Eu.Chol** (a) in HEPES buffer and its response to 10 and 20 µM of ONOO^−^ (pulse solution in 0.3M NaOH); in PBS buffer: (b) **Eu.Chol** response to 4, 8, 12 and 15 µM of ONOO⁻ (pulse solution in 0.3M NaOH); (c) **Eu.Chol** response to 24, 47, 70 and 92 µM of H_2_O_2_ (pulse solution in 0.3M NaOH); (d) **Eu.Chol** response to 70 and 136 µM of H_2_O_2_ (without NaOH); (e) **Eu.Chol** response to 8.7 and 17 mM of NaOH. Note, the final pH of the studies done with stock solutions containing 0.3M (NaOH) reaches around 11.

**Figure S26.** Time-resolved luminescence response of pre-incorporated **Eu.Chol** (1 mol%, 4 µM) in POPC liposomes. After 200 seconds, while the sample was stirring, a pulse of analytes was added to the liposomes and emission intensity at 615 nm was monitored for further 60 minutes. (a) in HEPES buffer cumulative addition of ONOO⁻ (3.5, 7, and 10 µM in 0.3 M NaOH) and (b) single additions of ONOO⁻ (12 and 22 µM in 0.3 M NaOH) in PBS buffer. Control experiments in PBS buffer were carried out with (c) 70 and 136 µM of H_2_O_2_ in 0.3 M NaOH and (d) 136 µM of H_2_O_2_ (without NaOH), 17 mM of NaOH (pulse solution of 0.3M NaOH) and 136 µM of H_2_O_2_ (pulse solution of H_2_O_2_ in 0.3M NaOH, making 17 mM of NaOH as global solution). The final pH of the studies can reach around 11 in the presence of 17 mM of NaOH. DLS was used to confirm the stability of liposomes at such pH.

**Figure S27.** Flow cytometry analysis of **Eu.Chol**-stained cells showing emission intensity distributions of (a) untreated cells and (b) cells treated with ONOO⁻ (100 μM) for 10 min. Panel (c) shows a histogram overlay comparing the emission intensity distributions of both cell populations. Eu(III) emission was collected using a 675/30 nm bandpass emission filter.

5. **References**
   1 C. Wang, D. Cheng, N. Jalali Motlagh, E. G. Kuellenberg, G. R. Wojtkiewicz, S. P. Schmidt, R. Stocker and J. W. Chen, *J. Med. Chem.*, **2021**, 64, 5874–5885.
   2 C. Breen, R. Pal, M. R. J. Elsegood, S. J. Teat, F. Iza, K. Wende, B. R. Buckley and S. J. Butler, *Chem. Sci.*, **2020**, 11, 3164–3170.
   3 S. E. Bodman, C. Breen, F. Plasser and S. J. Butler, *Org. Chem.Front.*, **2022,** 9, 5494–5504.
   4 S. Boonyarattanakalin, S. E. Martin, S. A. Dykstra and B. R. Peterson, *J. Am. Chem. Soc.*, **2004**, 126, 16379–16386.
